# Hemodynamic Responses Link Individual Differences in Informational Masking to the Vicinity of Superior Temporal Gyrus

**DOI:** 10.3389/fnins.2021.675326

**Published:** 2021-07-22

**Authors:** Min Zhang, Nima Alamatsaz, Antje Ihlefeld

**Affiliations:** ^1^Department of Biomedical Engineering, New Jersey Institute of Technology, Newark, NJ, United States; ^2^Rutgers Biomedical and Health Sciences, Rutgers University, Newark, NJ, United States

**Keywords:** informational masking, masking, auditory perception, functional near infrared spectrocopy, cochlear implant, hearing

## Abstract

Suppressing unwanted background sound is crucial for aural communication. A particularly disruptive type of background sound, informational masking (IM), often interferes in social settings. However, IM mechanisms are incompletely understood. At present, IM is identified operationally: when a target should be audible, based on suprathreshold target/masker energy ratios, yet cannot be heard because target-like background sound interferes. We here confirm that speech identification thresholds differ dramatically between low- vs. high-IM background sound. However, speech detection thresholds are comparable across the two conditions. Moreover, functional near infrared spectroscopy recordings show that task-evoked blood oxygenation changes near the superior temporal gyrus (STG) covary with behavioral speech detection performance for high-IM but not low-IM background sound, suggesting that the STG is part of an IM-dependent network. Moreover, listeners who are more vulnerable to IM show increased hemodynamic recruitment near STG, an effect that cannot be explained based on differences in task difficulty across low- vs. high-IM. In contrast, task-evoked responses near another auditory region of cortex, the caudal inferior frontal sulcus (cIFS), do not predict behavioral sensitivity, suggesting that the cIFS belongs to an IM-independent network. Results are consistent with the idea that cortical gating shapes individual vulnerability to IM.

## 1. Introduction

Perceptual interference from background sound, also called auditory masking, has long been known to impair the recognition of aurally presented speech through a combination of at least two mechanisms. Energetic masking (EM) occurs when target and masker have energy at the same time and frequency, such that the masker swamps or suppresses the auditory nerve activity evoked by the target (Young and Barta, 1986; Delgutte, 1990). Informational masking (IM) is presently defined operationally. IM occurs when a target is expected to be audible based on EM mechanisms, yet cannot be dissociated from the background sound. Listeners experience IM when the masker is target-like (e.g., hearing two women talk at the same time vs. hearing out a female in the background of a male voice; Brungart, 2001b) or when the listener is uncertain about perceptual features of the target or masker [e.g., trying to hear out a target with known vs. unexpected temporal patterning, cf. Lutfi et al. (2013)].

Unlike EM, IM is associated with striking variation in individual vulnerability (Neff and Dethlefs, 1995; Durlach et al., 2003). Moreover, an individual's susceptibility to IM is largely refractory to training (Neff et al., 1993; Oxenham et al., 2003). Identifying brain regions where IM-evoked activation patterns covary with individual differences in behavioral vulnerability to IM may thus hold a key for defining the neural mechanisms underlying IM.

Neuroimaging studies have greatly advanced our understanding of the neural mechanisms of masking. Converging evidence links both EM and IM to recruitment of superior temporal gyrus (STG) and frontal cortex (Davis and Johnsrude, 2003, 2007; Scott et al., 2004, 2006, 2009; Mesgarani and Chang, 2012; Lee et al., 2013; Michalka et al., 2015). For instance, the predominantly activated STG hemisphere can shift depending on the amount of IM in the background sound (Scott et al., 2009). Moreover, for speech that was either spectrally degraded or had impoverished amplitude cues, spanning the range from unintelligible to fully intelligible, activation near STG can account for approximately 40 to 50% of the variance in speech intelligibility (Pollonini et al., 2014; Lawrence et al., 2018).

In addition, lateral frontal cortex engages more strongly with increasing listening effort or increasing recruitment of higher-order semantic processes (Davis and Johnsrude, 2003; Scott et al., 2004; Wild et al., 2012; Wijayasiri et al., 2017). Parts of lateral frontal cortex, including the caudal inferior frontal sulcus (cIFS), are also sensitive to auditory short-term memory load in situations with IM (Michalka et al., 2015; Noyce et al., 2017). Using functional near-infrared spectroscopy (fNIRS), we previously confirmed that the cIFS region engages more strongly when listeners actively attend to speech in IM vs. listen passively (Zhang et al., 2018), making the STG and cIFS promising regions of interest (ROIs) for the current study.

Widening an established IM paradigm (Arbogast et al., 2002), we here compare hemodynamic responses to low vs. high IM speech. We test two hypotheses. H1: Individual differences in vulnerability to IM are mediated through processing limitations in the vicinity of STG. H2: Individual differences in vulnerability to IM arise near cIFS. Both hypotheses predict that for a given task difficulty, hemodynamic response strength in STG (H1) or cIFS (H2) accounts for behavioral sensitivity in situations where the background sound is target-like, but should not correlate with behavioral performance when the background sound is unlike the target.

To study how cortical responses shape individual differences in behavioral speech comprehension, our goal is to differentiate between brain areas with IM independence (task-evoked responses do not predict vulnerability to IM) vs. areas with IM dependence (task-evoked responses predict IM vulnerability). Using psychometric testing and fNIRS, we simultaneously quantify behavioral sensitivity and hemodynamic responses in the vicinity of STG and cIFS. In experiment 1, we contrast hemodynamic responses to speech detection in presence of combined target-unlike background noise (“low-IM”) vs. target-like background speech (“high-IM”). In both conditions, target and background sound are presented to both ears, resulting in same-ear masking. Low-IM vs. high-IM maskers have similar long-term spectral densities. Therefore, the amount of energetic masking is comparable across those conditions. To elucidate the role of EM, in experiment 2, we then contrast high-IM with same-ear vs. opposite-ear masking. The same-ear high-IM condition is similar to that of experiment 1. The two experiments serve as their own control, confirming test-retest reliability of the measured cortical traces. However, in the opposite-ear condition, target and high-IM never excite the same cochlea and therefore EM cannot occur. Our results support H1 but not H2.

## 2. Results

### 2.1. Experiment 1

**Figure 1:**
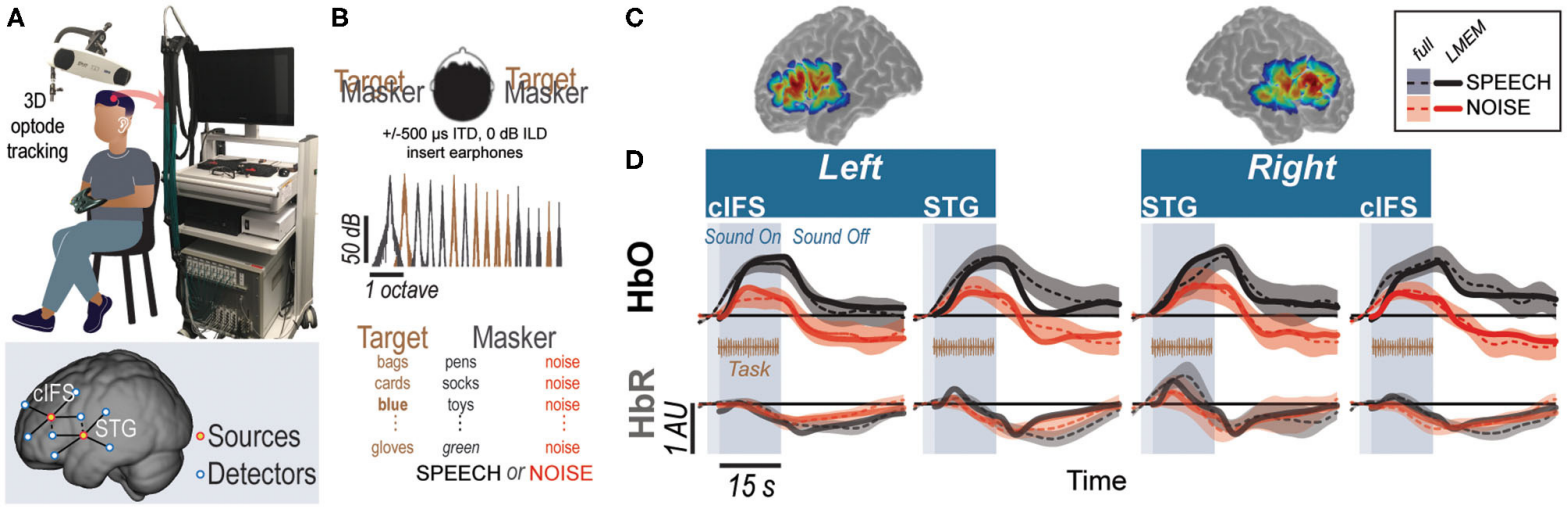
High-IM elicits stronger task-evoked responses than low-IM across all tested ROIs in experiment 1. **(A)** Experimental apparatus and setup and optode placement for a representative listener. Blue circles show placements of detector optodes, red circles of source optodes. Optodes are geometrically arranged into two types of source-detector channels: (1) deep recording channels and (2) shallow reference channels. Source-detector pairs of deep recording channels are separated by 3 cm (solid lines in the bottom insert). Source-detector pairs of reference channels are separated by 1.5 cm (dashed lines in the bottom insert). **(B)** Task design for SPEECH vs. NOISE. Both target (left-leading interaural time difference [ITD] of –500 *μs*) and masker (right-leading ITD of 500 *μs*) were presented binaurally. Spectral densities for target vs. masker show mutually flanking, sharply tuned component bands. **(C)** Sensitivity maps for optodes placed in the vicinity of STG and cIFS. Warmer colors denote increased likelihood that photons will be recorded from these areas. **(D)** HbO (top) and HbR (bottom) traces. Full hemodynamic responses are denoted by solid lines and error ribbons. Here and elsewhere, ribbons show one standard error of the mean across listeners. Task-evoked hemodynamic responses predicted from the linear mixed effects model (LMEM) are shown as dashed lines. Shaded areas mark the task duration.

Using the setup shown in Figure 1A, we recorded hemodynamic responses near cIFS and STG bilaterally, from normal-hearing young individuals. Target and masker were presented at equal broadband intensities to both ears. However, due to the presence of ITDs, listeners perceived the target as sounding from the left and the masker as sounding from the right. Listeners were instructed to detect when the target voice on the left uttered color keywords while SPEECH vs. NOISE maskers interfered from the right side (Figure 1B). Behavioral pilot testing confirmed that these spectrally sparse maskers produced high-IM (SPEECH) vs. low-IM (NOISE, Supplemental Information 1).

Accounting for approximately half of the variance in the recorded traces (*R*^2^ = 0.45), a single Linear Mixed Effects Model (LMEM; see Supplemental Information 2) was then used to predict task-evoked hemodynamic responses, by regressing out reference channels (β_6_ and β_7_), block number (β_5_), and pure-tone audiometric detection thresholds (PTA; β_11_ and β_12_) from the full response (Supplemental Information 2). Note that the reference channels comprise 44.6% of the total activation levels in the LMEM fits, as calculated via the area under the fitted curve with vs. without β_6_ and β_7_. Task-evoked responses were modeled by a canonical hemodynamic response function (HRF) and that function's first derivative (HRF') to improve temporal accuracy in the fit. Indeed, unlike the full hemodynamic response, the LMEM-estimated task-evoked hemodynamic response aligns well with the task-onset (compare onset of darker shaded area and dashed line throughout Figure 1D).

Our main interest was to determine the weights of the LMEM factors modeling cortical hemisphere, cortical structure, and masker configuration. LMEM fits reveal significant task-evoked responses at all four ROIs (Table 1; β_1−4_ > 0, *p* < 0.0001; see Figure 1D for HbO (top row) and HbR traces (bottom row). Moreover, all ROIs were sensitive to IM. Activation was stronger in the SPEECH as compared to the NOISE configuration (β_10_ > 0). The size of the difference between SPEECH (black lines in Figure 1D) vs. NOISE (red lines) activation varied across ROIs, but these interactions with ROI were small compared to the overall effect size (interaction between masker configuration and cortical structure: β_13_ < 0; interaction between masker configuration and hemisphere: β_14_ < 0; see Supplemental Information 3).

**Table 1:** Results of LMEM, experiment 1.

	**Term**		**Estimate**	**S.E**.	***t***	***p***	
β_0_	Intercept		–0.35	0.092	–3.8	0.0001	^***^
β_1_	HRF_*HbO*_		0.55	0.004	138.3	<0.0001	^***^
β_2_	HRF'_*HbO*_		0.17	0.004	39.4	<0.0001	^***^
β_3_	HRF_*HbR*_		0.02	0.004	5.8	<0.0001	^***^
β_4_	HRF'_*HbR*_		0.11	0.043	26.8	<0.0001	^***^
β_5_	Block number		0.01	0.000	76.6	<0.0001	^***^
β_6_	Reference channel_*HbO*_		0.42	0.000	1342.0	<0.0001	^***^
β_7_	Reference channel_*HbR*_		0.44	0.001	580.8	<0.0001	^***^
β_8_	Hemisphere		0.04	0.028	1.5	0.14	
β_9_	Cortical structure		0.08	0.026	3.0	0.003	^**^
β_10_	Masker		0.14	0.061	2.2	0.025	^*^
β_11_	R ear PTA		0.02	0.008	1.8	0.08	.
β_12_	L ear PTA		–0.01	0.005	–0.9	0.38	
β_13_	Masker configuration	: Cortical structure	–0.03	0.003	–12.8	<0.0001	^***^
β_14_	Masker configuration	: Hemisphere	–0.05	0.003	–21.1	<0.0001	^***^
β_15_	Cortical structure	: Hemisphere	–0.01	0.003	–5.4	<0.0001	^***^
β_16_	HRF_*HbO*_	: Masker configuration	–0.19	0.004	–46.5	<0.0001	^***^
β_17_	HRF_*HbO*_	: Cortical structure	0.17	0.004	41.6	<0.0001	^***^
β_18_	HRF_*HbO*_	: Hemisphere	-0.43	0.004	–10.8	<0.0001	^***^
β_19_	HRF'_*HbO*_	: Masker configuration	0.02	0.004	5.6	<0.0001	^***^
β_20_	HRF'_*HbO*_	: Cortical structure	–0.22	0.004	–51.6	<0.0001	^***^
β_21_	HRF'_*HbO*_	: Hemisphere	–0.04	0.004	–9.5	<0.0001	^***^
β_22_	HRF_*HbR*_	: Masker configuration	–0.12	0.004	–30.2	<0.0001	^***^
β_23_	HRF_*HbR*_	: Cortical structure	–0.01	0.004	–1.0	0.3	
β_24_	HRF_*HbR*_	: Hemisphere	0.05	0.004	11.9	<0.0001	^***^
β_25_	HRF'_*HbR*_	: Masker configuration	-0.10	0.004	–22.4	<0.0001	^***^
β_26_	HRF'_*HbR*_	: Cortical structure	0.16	0.004	36.6	<0.0001	^***^
β_27_	HRF'_*HbR*_	: Hemisphere	0.04	0.004	9.3	<0.0001	^***^

*Source: Zhang et al., 2021*.

*All estimates are referenced to a default condition in left cIFS for SPEECH*.

*Significance codes:*

*** *p < 0.001*,

** *p < 0.01*,

* *p < 0.05*,

*^.^ p < 0.1, p 0.1. Int, intercept; S.E., standard error of the mean*.

### 2.2. Experiment 2

The sharply tuned, mutually flanking bands of target and masker in experiment 1 were presented to both ears, and were designed to produce high- vs. low IM, with little EM. However, IM can also occur when target and masker are presented to opposite ears. It is unclear whether the neural mechanisms underlying IM are similar when target and masker are presented to the same vs. opposite ears. Thus, we next wished to examine whether the pattern of STG and cIFS recruitment would generalize to a dichotic IM configuration.

**Figure 2:**
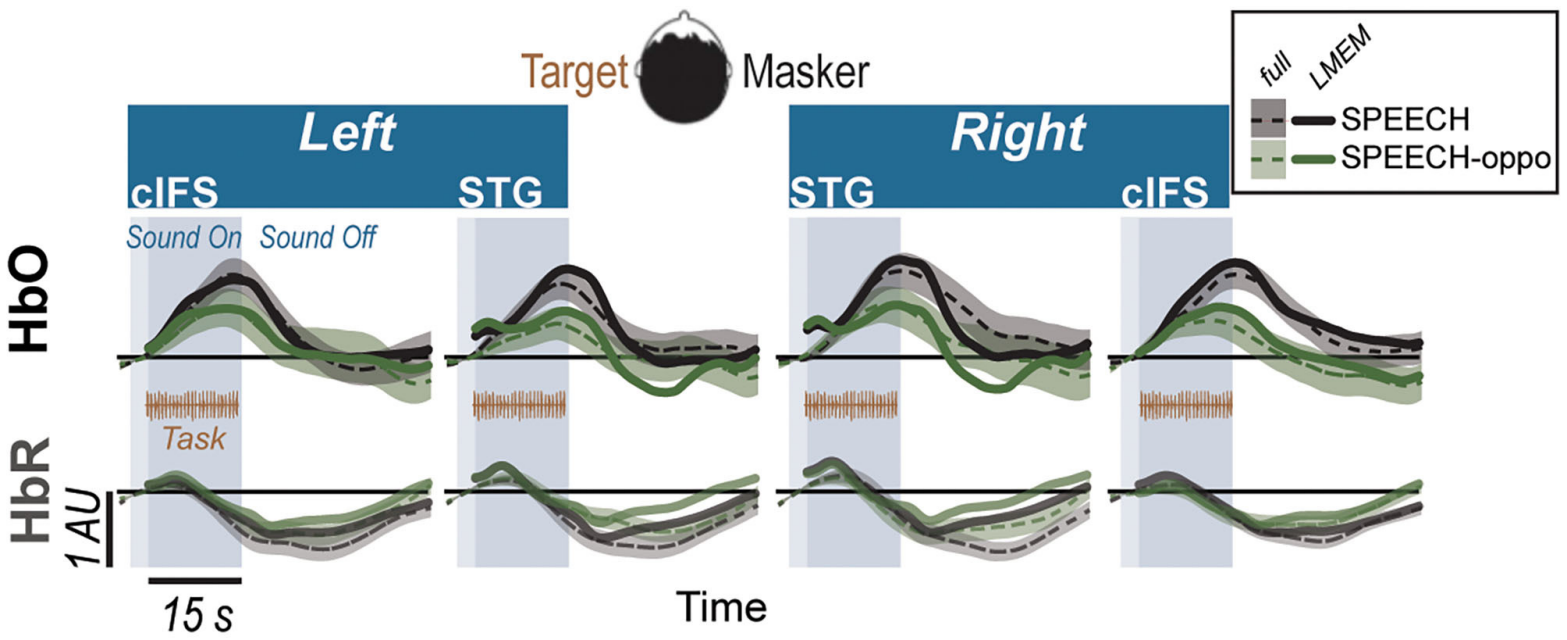
Hemodynamic responses for SPEECH (Black) vs. SPEECH-oppo (green) show robust task-evoked recruitment of all ROIs in experiment 2, even when target and masker are presented to opposite ears. Solid lines and error ribbons denote raw recordings; dashed lines show LMEM fits.

Testing a new group of 14 listeners, experiment 2 contrasted SPEECH with SPEECH-oppo, a stimulus configuration that was identical to SPEECH, except that target and masker were now presented to opposite ears (Figure 2). Mirroring results from experiment 1, a single LMEM fitting all HbO and HbR traces from experiment 2 accounted for approximately half of the variance in the recorded data (*R*^2^ = 0.52), with 60.2% of the full hemodynamic activation attributed to reference channels. Moreover, LMEM fits confirmed that task-evoked responses in all four ROIs occurred in both masker configurations, even when target and masker were presented to opposite ears (Table 2; β_1−4_ > 0, *p* < 0.0001). All ROIs engaged more strongly in the SPEECH as compared to the SPEECH-oppo configuration (β_10_ > 0), with effect size depending somewhat on ROI (see Supplemental Information 3).

**Table 2:** Results of LMEM, experiment 2.

	**Term**		**Estimate**	**S.E**.	***t***	***p***	
β_0_	Intercept		0.02	0.065	0.3	0.75	
β_1_	HRF_*HbO*_		0.29	0.003	89.4	<0.0001	^***^
β_2_	HRF'_*HbO*_		0.07	0.003	19.5	<0.0001	^***^
β_3_	HRF_*HbR*_		–0.04	0.003	–10.9	<0.0001	^***^
β_4_	HRF'_*HbR*_		0.07	0.004	20.8	<0.0001	^***^
β_5_	Block number		0.01	0.000	39.1	<0.0001	^***^
β_6_	Reference channel_*HbO*_		0.67	0.001	1490.4	<0.0001	^***^
β_7_	Reference channel_*HbR*_		0.73	0.001	802.0	<0.0001	^***^
β_8_	Hemisphere		–0.02	0.025	–0.7	0.46	
β_9_	Cortical structure		0.04	0.034	1.2	0.23	
β_10_	Masker		0.00	0.025	0.04	0.97	
β_11_	R ear PTA		–0.01	0.011	–0.97	0.33	
β_12_	L ear PTA		0.00	0.009	0.3	0.79	
β_13_	Masker configuration	: Cortical structure	0.06	0.002	26.3	<0.0001	^***^
β_14_	Masker configuration	: Hemisphere	–0.03	0.00	–14.5	<0.0001	^***^
β_15_	Cortical structure	: Hemisphere	0.08	0.002	40.3	<0.0001	^***^
β_16_	HRF_*HbO*_	: Masker configuration	–0.1	0.003	–31.8	<0.0001	^***^
β_17_	HRF_*HbO*_	: Cortical structure	0.04	0.003	11.1	<0.0001	^***^
β_18_	HRF_*HbO*_	: Hemisphere	0.03	0.003	8.5	<0.0001	^***^
β_19_	HRF'_*HbO*_	: Masker configuration	–0.01	0.003	–1.8	0.072	.
β_20_	HRF'_*HbO*_	: Cortical structure	–0.19	0.003	–53.9	<0.0001	^***^
β_21_	HRF'_*HbO*_	: Hemisphere	–0.06	0.003	–16.63	<0.0001	^***^
β_22_	HRF_*HbR*_	: Masker configuration	0.003	0.003	1.1	0.29	
β_23_	HRF_*HbR*_	: Cortical structure	–0.05	0.003	–14.4	<0.0001	^***^
β_24_	HRF_*HbR*_	: Hemisphere	–0.04	0.003	–11.9	<0.0001	^***^
β_25_	HRF'_*HbR*_	: Masker configuration	0.01	0.003	3.0	0.0031	^**^
β_26_	HRF'_*HbR*_	: Cortical structure	0.06	0.003	17.8	<0.0001	^***^
β_27_	HRF'_*HbR*_	: Hemisphere	–0.01	0.003	–3.5	0.0006	^***^

*Source: Zhang et al., 2021*.

*All estimates are referenced to a default condition in left cIFS for SPEECH. Significance codes:*

*** *p < 0.001*,

** *p < 0.01, and ^.^ p < 0.1, p 0.1. Int, intercept; S.E., standard error of the mean. Int, intercept; S.E., standard error of the mean*.

### 2.3. Vulnerability to Masking and Hemodynamic Responses

To test the core hypotheses, we next examined STG and cIFS for IM-dependence. We reasoned that in an IM-dependent ROI, the hemodynamic activation strength should predict behavioral sensitivity. Specifically, should hemodynamic activation near an ROI predict behavioral sensitivity for high-IM but not low-IM this would support the idea that brain regions in the vicinity of that ROI are IM-dependent (H1: STG, H2: ciFS).

**Figure 3:**
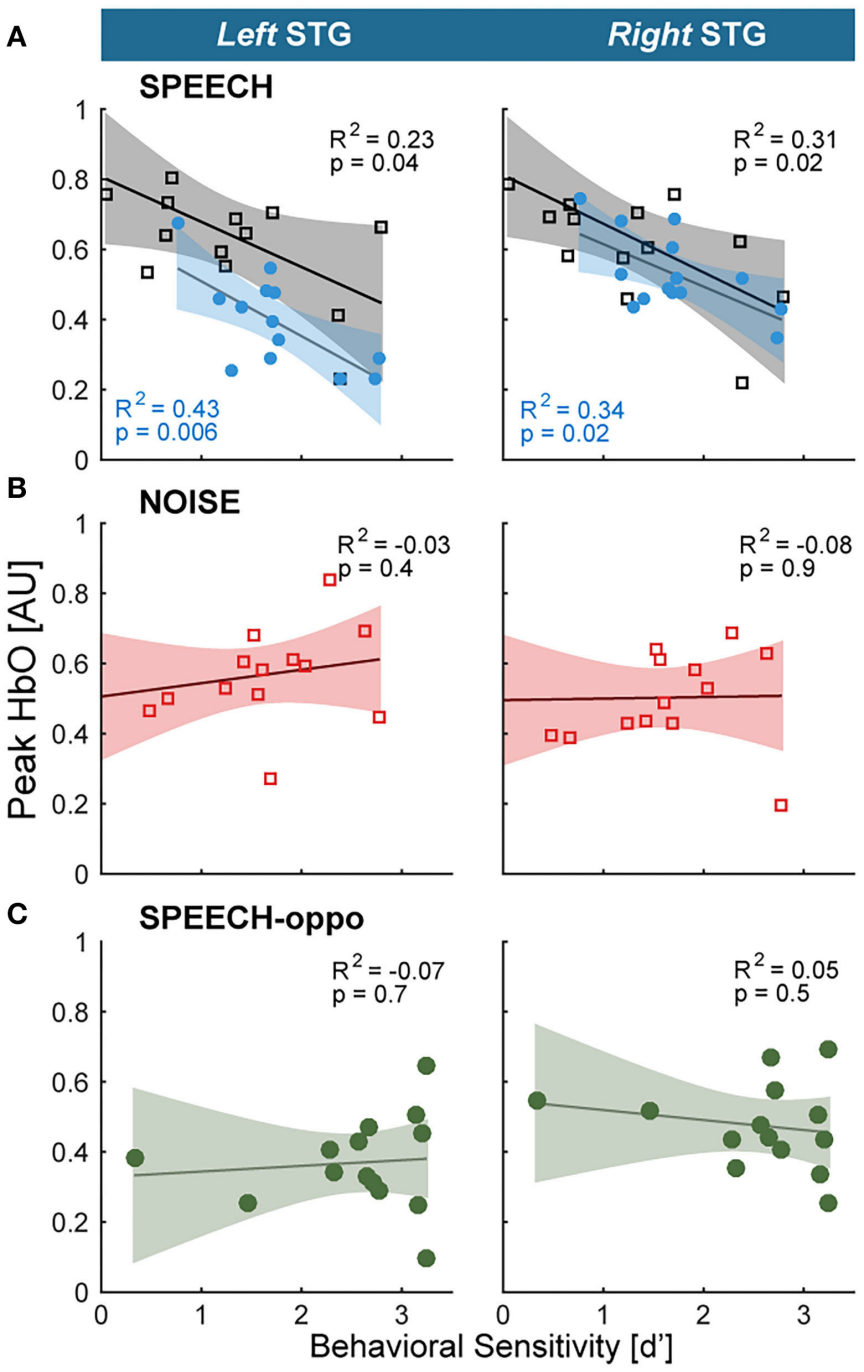
Hemodynamic responses link individual differences in vulnerability toward IM to the vicinity of STG **(A)** STG activity and behavioral vulnerability to the high-IM SPEECH condition are robustly anti-correlated, across both hemispheres in experiments 1 and 2 (black vs. blue symbols, respectively). **(B)** There was no appreciable association between HbO peaks and the low-IM NOISE condition. **(C)** When target and masker were presented to opposite ears in the SPEECH-oppo configuration, HbO peaks did not predict psychophysical thresholds.

For each ROI, planned adjusted coefficients of determination, *R*^2^, between behavioral speech detection sensitivity and the peak of the HbO response were calculated. In experiment 1, individual behavioral thresholds were significantly anti-correlated with peak HbO only in the SPEECH configuration in the vicinity of left or right STG, where hemodynamic responses explained 23% (left STG) and 31% (right STG) of the behavioral variance (black square symbols in Figure 3A). In contrast, behavioral NOISE thresholds were uncorrelated with hemodynamic responses (Figure 3B). Note that these differences in hemodynamic activation patterns were observed despite the fact that the behavioral speech detection performance, measured during the fNIRS recordings, was comparable between NOISE and SPEECH [paired *t*-test: *t*(13) = −1.14, *p* = 0.27]. Furthermore, activity levels near cIFS (Figure 1C) were not correlated with behavioral thresholds in SPEECH or NOISE.

Testing a different group of listeners, experiment 2 confirmed the finding from experiment 1 that HbO peaks near left or right STG were significantly anti-correlated with behavioral sensitivity for the SPEECH configuration. Moreover, activity levels in cIFS were again uncorrelated with behavioral thresholds. Identical SPEECH configurations were assessed in experiments 1 and 2. Therefore, the converging results across two groups of listeners confirm high test-retest reliability of the current fNIRS approach. Specifically, in experiment 2, STG HbO peak activation explained 43 and 34% of the behavioral variance in left and right STG, respectively, (blue square symbols in Figure 3A). In contrast, hemodynamic responses for SPEECH-oppo did not predict behavioral sensitivity (Figure 3C).

A caveat, unlike in experiment 1, in experiment 2, task difficulty differed across masking conditions. Specifically, behavioral speech detection thresholds were better for SPEECH-oppo than SPEECH [paired *t*-test: *t*_(13)_ = –3.13, *p* = 0.008; compare green symbols in Figure 3C falling to the right of the red, blue and black symbols in Figures 3A,B]. However, even for the more poorly performing listeners in experiment 2, no obvious trend links behavioral sensitivity to peak HbO levels in left or right STG.

Of note, behavioral responses were not predicted from HbR activity levels, across any of the tested conditions, in either of the two experiments. As expected, task-evoked HbO and HbR responses were robustly anti-correlated (in Figures 1D, 2, compare dark dashed lines in the top row to the lighter dashed lines of the same color in the bottom row). This anti-correlation would predict that HbR responses mirror the correlation patterns between HbO peaks and behavioral sensitivity. However, in general, HbR response magnitudes were very small, approximately 20% of HbO magnitudes, hinting that here, the HbR responses may have been contaminated by the noise floor of the recording system.

## 3. Discussion

The goal of the current work was to identify brain regions where individual differences in IM vulnerability emerge. To that end, we sought to differentiate between IM-independent parts of the brain whose activation levels are equivalently driven by low- or high-IM, vs. IM-dependent regions whose activation levels correlate with individual IM-vulnerability.

### 3.1. Hemodynamic Correlates of IM

The current data confirm that cortical regions at or near STG and cIFS engage during masked speech comprehension tasks (Scott et al., 2004, 2006, 2009; Kerlin et al., 2010; Ding and Simon, 2012; Mesgarani and Chang, 2012; Michalka et al., 2015; Noyce et al., 2017; Rowland et al., 2018; Zhang et al., 2018). For both high- and low-IM background sound, when a listener engaged in speech detection, robust task-evoked hemodynamic responses in STG and cIFS occurred in both brain hemispheres. Task-evoked bilateral responses in STG and cIFS were even observed when target and high-IM masker were presented to opposite ears (SPEECH-oppo in experiment 2).

SPEECH masking recruited a stronger task-evoked response than NOISE masking in both left and right STG, consistent with prior work (Scott et al., 2004). Activation levels during SPEECH masking consistently predicted a moderate 30% of variability of individual differences in vulnerability in left or right STG, in both experiments. Moreover, STG recruitment did not predict vulnerability to masking for the low-IM masker (NOISE condition in experiment 1). Together, these results show that recruitment in the vicinity of STG was IM-dependent. In contrast, while cIFS also showed task-evoked responses that were stronger in SPEECH than in NOISE, cIFS activation strength did not significantly correlate with individual vulnerability in any tested masking configuration, suggesting that the vicinity of cIFS was IM-independent. The observed association between hemodynamic response recruitment near STG was somewhat greater in experiment 2 than in experiment 1, and more variable in left than right STG, hinting that an uncontrolled source of variance contributed. It is important to note that here, we did not systematically control for across-participant variability in skull curvature, skin pigmentation or hair coarseness across participants.

IM is thought to be a central auditory mechanism. However, IM generally interferes much more strongly when target and masker are presented to the same ear(s), as compared to being presented to opposite ears (Brungart and Simpson, 2002, 2007; Kidd Jr et al., 2003; Gallun et al., 2005; Wightman and Kistler, 2005). It is unclear whether these mechanisms are similar for same-ear vs. opposite ear IM. Even when background sound enters a non-target ear, behavioral evidence suggests that IM interference can be attributed to a combination of a failure to attend to the target ear as well as increased listening effort (Gallun et al., 2007), whereas same-ear masking adds the possibility that energetic masking shapes IM through interactions with attention and across-time streaming (Ihlefeld and Shinn-Cunningham, 2008).

Here, SPEECH-oppo evoked bilateral responses in STG and cIFS. If identical STG-based networks were activated for same-ear-IM (SPEECH) and opposite-ear-IM (SPEECH-oppo), STG activity should have been a negative predictor of behavioral SPEECH-oppo sensitivity, but this was not observed. Behavioral sensitivity in this task was derived by calculating the d' difference between the rate of correct button-press responses vs. the rate of false-alarm button-press responses one would have obtained had the participant pushed the response button equally often but randomly (see Methods and Materials), resulting in a theoretical maximum d' of 3.25. Note that speech identification thresholds in SPEECH-oppo were at or close to this psychometric ceiling for a few of the listeners (note clustering of five green points at the right of Figure 3C), biasing the regression fits toward zero slope. However, ignoring these high-performing listeners, even for poorly performing listeners, no trend emerged linking the peak HbO response and behavioral sensitivity (Figure 3C). Moreover, the interpretation that contralateral IM recruits different brain networks than ipsilateral IM is also supported by prior evidence from research in children, where the ability to suppress a masker ipsilateral to the target matures more slowly than the ability to suppress a masker on the contralateral side (Wightman et al., 2010).

For same-ear IM, listeners reached comparable speech detection thresholds in low-IM and high-IM, but had marked individual difference during IM speech identification during behavioral pilot testing. This observation is consistent with the idea that more IM-vulnerable listeners exerted more listening effort (Pichora-Fuller et al., 2016). A cortical marker for listening effort was previously located in lateral inferior frontal gyrus, a brain area which shows attention-dependent increase in frontal brain activation during listening to degraded speech (Wild et al., 2012; Wijayasiri et al., 2017). The current study did not target the lateral inferior frontal gyrus, nor did we record alternative measures of listening effort, such as pupilometry (Zekveld and Kramer, 2014; Parthasarathy et al., 2020), precluding any direct test of this possibility.

Together, the results show that even with comparable behavioral sensitivities and similar long-term acoustic energy, high-IM in the same ear increased HbO peaks near STG and cIFS, as compared to low-IM. This effect was observed separately for same-ear as well as opposite-ear IM. Moreover, the observed anti-correlation between HbO peak levels and individual task performance in same-ear high-IM is consistent with the interpretation that left and right STG are part of a same-ear-IM-dependent network. In contrast, the vicinity of cIFS engaged in an IM-independent manner.

### 3.2. Emergence of IM

Listeners with higher cognitive abilities comprehend masked speech better (Rönnberg et al., 2008; Mattys et al., 2012), but prior work shows no evidence that cognitive ability contributes differently to IM vs. EM. For instance, cognitive scores poorly predict how well an individual can utilize an auditory scene analysis cue to suppress IM (Füllgrabe et al., 2015). Consistent with this, here, task-evoked responses near cIFS were IM-independent, unlike in the vicinity of STG.

Inded, prior work hints that IM emerges at the level of auditory cortex, a part of the STG (Gutschalk et al., 2008). We here tested maskers that were spectrally interleaved with the target, designed to produce either high IM (SPEECH) or low IM (NOISE). EM, when present, was limited to spectral regions outside the frequency bands that comprised most of the target energy. Consistent with this, for speech *detection*, behavioral thresholds were comparable between SPEECH and NOISE. However, our behavioral pilot results also confirmed that speech *identification* was much more difficult in the presence of SPEECH than NOISE (Freyman et al., 1999; Arbogast et al., 2002; Brungart et al., 2006; Wightman et al., 2010).

This behavioral pattern parallels a behavioral phenomenon in vision—called Crowding. In Crowding, the presence of visual target identification is severely impaired by nearby clutter or “flankers” (Bouma, 1970; Rosen et al., 2014). In the current IM design, the spectrally sparse masker and target can be conceptualized as mutually flanking each other. Moreover, analogous to the current behavioral results, flankers that Crowd target identification do not affect target detection (Pelli et al., 2001). Furthermore, using a behavioral paradigm that is comparable to the current speech identification task, prior work shows that IM can occur even when the masker is softer than the target (Brungart, 2001a; Ihlefeld and Shinn-Cunningham, 2008). Analogously, Crowding can occur even when the flankers are smaller than the target (Pelli et al., 2001). Of importance to the current work, there is good evidence that the Crowding effect occurs in the visual cortex (Millin et al., 2014; Zhou et al., 2018a). In particular, flankers presented through one eye crowd a target presented through the other eye (Flom et al., 1963; Taylor and Brown, 1972; Tripathy and Levi, 1994). These striking similarities of IM and Crowding suggest that they result from analogous sensory processes, further supporting the prior notion that IM arises at the level of cortex.

### 3.3. Cortical Mechanisms of IM

The current results show that for similar behavioral sensitivities and similar long-term acoustic energy, individual differences in vulnerability to high-IM in the same ear correlated with increased need for supply of oxygen in the vicinity of STG, as compared to low-IM. However, converging evidence from prior work with electroencephalography (EEG) recordings also shows that the temporal fidelity by which cortical local field potentials encode sound, as opposed to their absolute response strength, correlates with task demands and predicts masked speech intelligibility (Choi et al., 2014; O'Sullivan et al., 2015; Viswanathan et al., 2019). Note that unlike with hemodynamic responses recorded with fNIRS, which emerge within proximity of the recording sensors at STG, it is generally more difficult to pinpoint where in the brain the EEG traces originate. In addition, even listeners with audiologically normally hearing can vary dramatically in their ability to resolve and utilize temporal fine structure cues (Ruggles et al., 2011; Bharadwaj et al., 2019). Moreover, an individual's sensitivity to monaural or binaural temporal fine structure predicts masked speech intelligibility, especially in temporally fluctuating background sound (Lorenzi et al., 2006; Papesh et al., 2017). Intriguingly, the neural mechanisms shaping temporal fidelity are thought to be of *sub*cortical origin (Parthasarathy et al., 2020). Furthermore, prior work with MEG indicates that a thalamo-cortical loop gates temporal signatures of sound to the cortical processing level (Bharadwaj et al., 2016). Consistent with this, recent cortical recordings in humans also demonstrate that neural tuning properties of the STG rapidly and flexibly shift in gain, temporal sensitivy and spectrotemporal tuning, depending on the stimulus (Khalighinejad et al., 2019; Keshishian et al., 2020).

Together, these findings raise the possibility that an individual's need for gating or adapting the neural code in STG should increase with decreasing temporal fidelity of subcortical information, as they need to work harder to overcome poor subcortical encoding of the target. Increased inhibitory activity in STG associated with stronger modulation or gating of subcortical temporal fidelity in vulnerable listeners should therefore increase the amplitude of hemodynamic responses (Stefanovic et al., 2004; Vazquez et al., 2018). Broadly increased inhibition would not necessarily be picked up via EEG analysis looking for temporal coherence and/or EEG recordings summing neural activity farther from STG. Thus, the current results are consistent the idea that increased gating or modulation of subcortical information via STG may be a potential mechanism contributing for individual variability in IM vulnerability. Future work is needed to explore how metabolic need and the fidelity of cortical temporal coding interact.

### 3.4. Spatial Specificity

The spacing of fNIRS optodes determines both the depth of the brain where recorded traces originate, as well as their spatial resolution along the surface of the skull. Here, optode sources and detectors were spaced 3 cm apart and arranged cross-wise around the center of each ROI (Figure 1A). To estimate the hemodynamic activity in each ROI, we averaged across the four channels of each ROI. This averaging greatly improved test-retest reliability of each ROI's activation trace during pilot testing, both here and in our prior work (Zhang et al., 2018). A caveat of this approach is that it reduces the spatial resolution of the recordings. Thus, it is unclear whether increased hemodynamic activity near STG is due to increased STG recruitment, or due to a more broadly activated brain network in the vicinity of STG. For instance, there is precedence for activation of additional brain regions as a compensatory strategy for coping with age-related cognitive decline (Presacco et al., 2016; Jamadar, 2020). Listeners who are more vulnerable may use either a broadened brain network or increase STG recruitment, two possibilities that the current data cannot differentiate. However, either interpretations is consistent with the idea that a central processing limitation exists that includes STG and shapes vulnerability to IM.

### 3.5. Diagnostic Utility

The current results bear clinical relevance. A technique we here used to design our stimuli, vocoding, is a core principle of speech processing with current cochlear implants. A pressing issue for the majority of cochlear implant users is that they cannot hear well in situations with masking, an impairment in part attributed to cortical dysfunction (Anderson et al., 2017; Zhou et al., 2018b). Sending target and masker sound to opposite ears can improve target speech identification in some, but not all, bilateral cochlear implant users of comparable etiology, suggesting that central auditory processing contributes to clinical performance outcomes (Goupell et al., 2016). This makes it desirable to assess auditory brain health in cochlear implant users. However, a challenge for imaging central auditory function in cochlear implant users is that cochlear implants are ferromagnetic devices. Thus, cochlear implants often either unsafe for use in magnetic resonance imaging (MRI) scanners and/or cause sizeable artifacts when imaged with MRI or EEG (Hofmann and Wouters, 2010). Moreover, when imaged under anesthesia, cochlear implant stimulation can fail to elicit cortical responses, making it potentially impractical to record cortical responses during CI surgeries (Nourski et al., 2013). In contrast, fNIRS, a quiet and light-based technology, is safe to use with cochlear implants. Albeit limited to a small number of participants, the current paradigm demonstrates feasibility: fNIRS-recorded cortical responses to masked speech with impoverished, cochlear-implant-like qualities, can explain approximately a third of the variance in individual vulnerability to IM–an approach that, it is hoped, may prove useful in future clinical practice.

## 4. Methods and Materials

### 4.1. Participants

Our sample size (14 participants for each of the two fNIRS experiments and 11 participants for a behavioral pilot control) was selected *a priori* using effect size estimates from prior work on IM (Arbogast et al., 2002; Zhang et al., 2018). Briefly, using prior psychometric functions of IM sensitivity, a sample size of 8 participants suffices to demonstrate behavioral differences in the task conditions tested here (Arbogast et al., 2002; Brungart and Simpson, 2007; Ihlefeld and Shinn-Cunningham, 2008). For the fNRIS recordings, where prior data with the specific recording system and auditory task did not exist, we ran a bootstrapping analysis, sampling with replacement our prior recordings on a related task (Zhang et al., 2018). We needed at least 12 participants to reliably arrive at the effect size that we previously observed with 10% tolerance (Zhang et al., 2018). We then conservatively chose slightly more participants than we had estimated. In total, we recruited 40 paid listeners, who were right-handed native speakers of English, and between 19 and 25 years old (17 females). Assessment of pure-tone audiometric detection thresholds (PTAs) at all octave frequencies from 250 to 8 kHz of 20 dB HL or better verified that all listeners had normal hearing. Specifically, the across-ear differences in pure tone thresholds was 10 dB or less, at all of the audiometric frequencies. All listeners gave written informed consent prior to participating in the study. All testing was administered according to the guidelines of the Institutional Review Board of the New Jersey Institute of Technology.

### 4.2. Speech Stimuli

There were 16 possible English words, each utterance recorded without co-articulation by each of two male talkers (Kidd Jr et al., 2008). The words consisted of the colors <red, white, blue, and green> and the objects <hats, bags, cards, chairs, desks, gloves, pens, shoes, socks, spoons, tables, and toys>. The colors were designated as keywords. Target word sequences were generated by picking a total of 25 random words from the overall set of 16, including between three and five target words, and concatenating them in random order with replacement (a set of more than 10^26^ possible permutations for the target sequence, $(\frac{27}{3})\cdot 12^{22}\cdot 4^{3}+(\frac{28}{4})\cdot 12^{21}\cdot 4^{4}+(\frac{29}{5})\cdot 12^{20}\cdot 4^{5}>1.6\cdot 10^{16}$). Similarly, masker sequences were made by picking 25 random words from the overall set of 16, constrained such that target and masker words always differed from each other, for any given word position in the target and masker sequence. One talker was used for the target, the other for the masker. Prior to concatenation, each utterance was initially time-scaled to a duration of 300 ms (Hejna and Musicus, 1991). In addition, 300 ms silences were included between consecutive words, such that the total duration of each target sequence equaled 15 s.

### 4.3. Vocoding

Next, the target word sequences were vocoded through an analysis-, followed by a synthesis-filtering stage. For the analysis stage, each word sequence was filtered into 16 adjacent spectral bands, with center frequencies from 300 to 10 kHz. These spectral bands were spaced linearly along the cochlea according to Greenwood's scale, with a distance of more than one equivalent rectangular cochlear bandwidth between neighboring filters (Greenwood, 1990; Chen et al., 2011). Analysis filters had a simulated spectral width of 0.37 mm along the cochlea (Greenwood, 1990) or approximately 1/10th octave bandwidth, had a 72 dB/octave frequency roll-off and were implemented via time reversal filtering, resulting in zero-phase distortion. In each narrow speech band, the temporal envelope of that band was then extracted using Hilbert transform. Broadband uniformly distributed white noise carriers were multiplied by these envelopes. For the synthesis stage, these amplitude-modulated noises were then processed by the same filters that were used in the analysis stage. Depending on the experimental condition, a subset of these 16 bands was then added, generating an intelligible, spectrally sparse, vocoded target sequence.

### 4.4. Target/Masker Configurations

A target sequence was always presented simultaneously with a masker sequence. Analogous to an established behavioral paradigm for assessing IM, we used two different masker configurations, consisting of different-band-speech or different-band-noise (Arbogast et al., 2002). In the SPEECH condition, the masker sequence was designed similarly to the target except that it was constrained such that (1) the target and masker words were never equal at the same time and (2) the masker was constructed by adding the remaining seven spectral bands not used to build the target sequence. In the NOISE condition, the masker sequence consisted of 300-ms long narrowband noise bursts that were centered at the seven spectral bands not used to build the target sequence. All processing steps were identical to the SPEECH condition, expect that, instead of being multiplied with the Hilbert envelopes of the masker words, the noise carriers were multiplied by 300-ms long constant-amplitude envelopes that were ramped on and off with the target words (10 ms cosine squared ramps). Figure 1A shows a representative spectral energy profile for a mixture of target (brown) and SPEECH (black) sequences. Note that the spectrum of a mixture of target and NOISE samples comprised of similar frequency bands would look visually indistinguishable from target in SPEECH and is thus not shown here (c.f. Arbogast et al., 2002).

In experiment 1, target and either a different-band speech or a different-band-noise masker were presented binaurally (Figure 1B). The target had a left-leading interaural time difference (ITD) of -500 *μs*. The masker sequence had a right-leading 500 *μs* ITD, resulting in two possible target/masker configurations, called SPEECH (different-band-speech with 500 *μs* ITD) vs. NOISE (different-band-noise with 500 *μs* ITD). The target and masker were each presented at 59 dBA, as calibrated with a 1-kHz tone that was presented at the same root mean square as the target and masker and recorded with KEMAR microphones (Knowles Electronics model KEMAR 45BB). As a result, the broadband Target-to-masker energy ratio (TMR) equaled 0 dB. However, at each of the center frequencies of the nine vocoded spectral bands that made up the target, the TMR equaled 93 dB or more.

In experiment 2, the masker always consisted of a different-band-speech sequence. Target and masker sequences were presented in two possible configurations. The first configuration was identical to the SPEECH condition of experiment 1, with the target presented binaurally with a –500 *μs* ITD and a SPEECH masker at 500 *μs* ITD. In the second “SPEECH-oppo” configuration, a target and different-band-speech masker were presented to opposite ears, with the target presented monaurally to the left, and a different-band-speech masker monaurally to the right ear (Figure 2).

### 4.5. Behavioral Task

The auditory task consisted of 12 45-s long blocks. To familiarize the listener with the target voice, at the beginning of each block, we presented a 3-s long cue sentence with the target talker's voice and instructed the listeners to direct their attention to this talker. The cue sentence was “Bob found five small cards,” and was processed identically to the target speech for that block (same spectral bands, same binaural configuration). Each block then consisted of a 15-s long acoustic mixture of one randomly generated target and one randomly generated masker sequence, followed by a rest period of 30 s of silence. Moreover, at the end of each auditory task block, we added a random silent interval (mean: 3.8 s, variance: 0.23 s, uniform distribution). In experiment 1, we randomly interleaved six SPEECH blocks with six NOISE blocks, whereas in experiment 2, we randomly interleaved six SPEECH blocks with six SPEECH-oppo blocks. The spectral bands of the vocoded target and masker were fixed within each block and randomly interleaved across blocks.

Listeners were instructed to press a button each time the target talker to their left side uttered any of the four color keywords, while ignoring all other words from both the target and the masker. A random number (between three and five) of color words in the target voice would appear during each block. No response feedback was provided to the listener.

### 4.6. Behavioral Detection Threshold

Throughout each block we counted *N*_*B*_, the number of intervals that the listener pushed the button of the response interface. If a button push occurred within 200–600 ms after the onset of a target keyword, the response was scored as a hit. Absence of any button push response in the same time period was scored as a miss. The observed percent correct was calculated by dividing the number of hits by the total number of target keywords during that block.

The baseline guessing rate was estimated via a bootstrapping analysis that calculated the chance percent correct that a simulated listener would have obtained by randomly pushing a button N times throughout that block. Specifically, to estimate the chance percent of keywords guessed correctly via random button push, for each particular listener and block, we randomly shuffled *N*_*B*_ button push intervals across the duration of that particular block's target sequence and counted the number of keywords guessed correctly, then repeated the process by randomly shuffling again for a total of 100 repetitions. To correct for bias, the observed vs. chance percent correct scores were then converted to d'-scores, by calculating the difference in z-scores of observed percent correct vs. chance percent correct (Klein, 2001). To prevent infinite d' values, hit and guessing rates were bracketed such that they could not fall below 0.001 and could not exceed 0.999.

### 4.7. Behavioral Pilot Control

Behavioral pilot testing established the presence of IM in our stimuli, while also verifying that the high- vs. low-IM conditions tested *via* fNIRS resulted in comparable speech intelligibility. Inside a double-walled sound-attenuating booth (Industrial Acoustic Company), we tested 11 normal-hearing listeners using the same auditory testing equipment and the same speech detection task that we used during the fNIRS recordings, except that listeners had their eyes open during this pilot testing.

In addition, using vocoded stimuli that were recorded by the same talkers as the stimuli used for the speech detection task, we assessed speech identification thresholds by using the coordinate response measure task (Brungart, 2001b; Kidd Jr et al., 2008). Briefly, this task presents listeners with the following sentence structure: “Ready [call sign] go to [color] [number] now.” There were eight possible call signs < Arrow, Baron, Charlie, Eagle, Hopper, Laker, Ringo, Tiger>, the same four colors as in the detection task <red, blue, white, green>, and seven numbers (numbers one through eight, except “seven” because, unlike the other numbers, it consists of two syllables). The target sentence was spoken by the same talker for every trial and always had “Baron” as call sign; the masker was either SPEECH or NOISE from a different talker, and using a different call sign than “Baron.” Listeners were instructed to answer the question “Where did Baron go?” by identifying the color in the target sentence. The masker was held fixed at 65 dB SPL, whereas the target level varied randomly from trial to trial from 45 to 85 dB SPL, resulting in five possible TMRs from –20, –10, 0, 10, and 20 dB. The target levels were randomized such that all five TMRs were tested in random order before all of them were repeated in different random order. Listeners competed 20 trials per TMR, both in SPEECH and in NOISE. In addition, to verify that all listeners could understand the vocoded speech in quiet at the softest target level, prior to testing masked thresholds, listeners completed 20 trials in quiet at 45 dB SPL.

**Figure 4:**
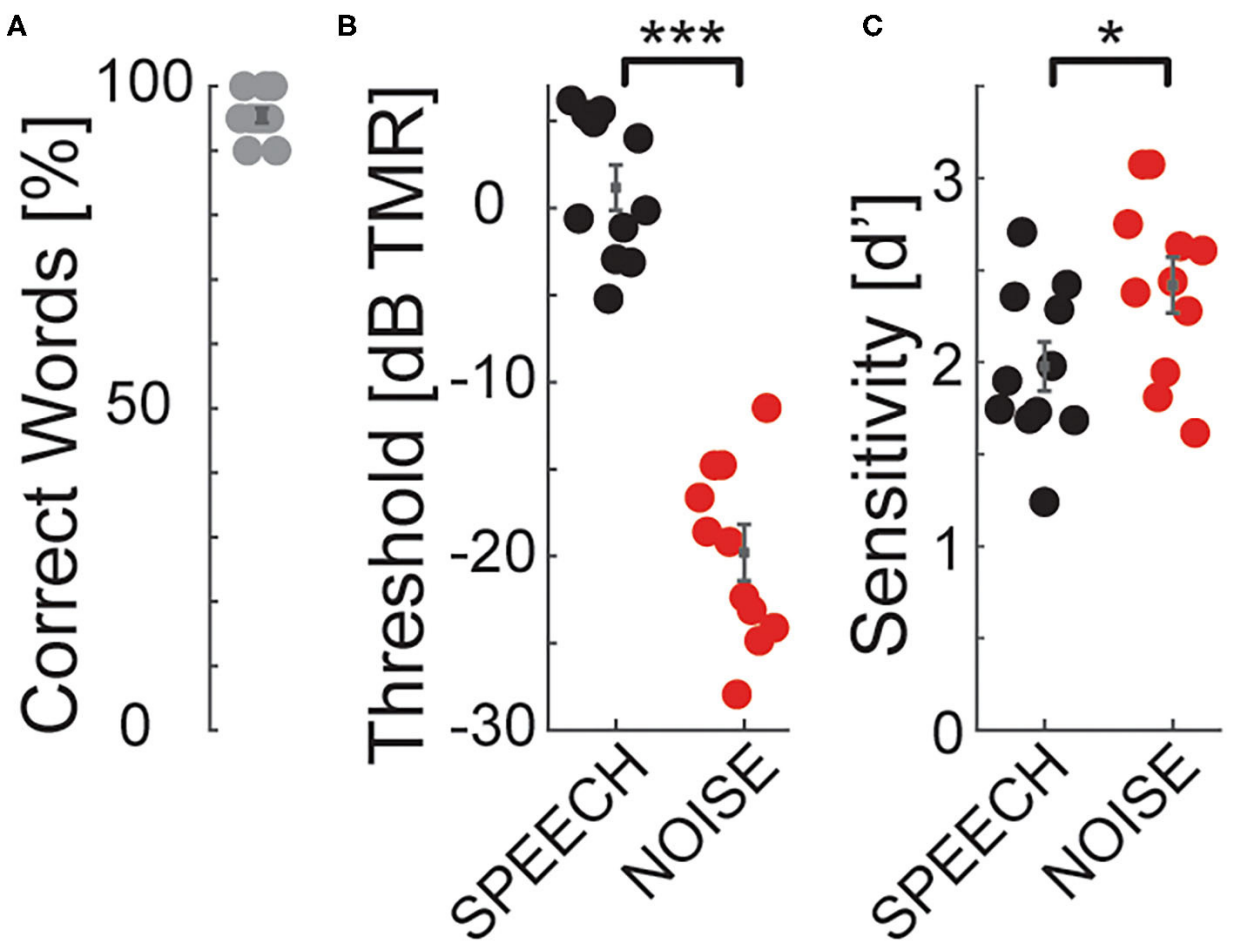
Speech identification and detection performance during pilot testing for SPEECH vs. NOISE confirm that the SPEECH masker causes IM. The target had a left-leading ITD of -500's; the masker a right-leading ITD of 500 μs. **(A)** Quiet thresholds. Percent correct keywords identified without masker. **(B)** Speech identification task. Percent correct keywords identified with SPEECH (black) or NOISE (red) masking. **(C)** Speech detection task. Sensitivity to keywords with SPEECH (black) or NOISE (red) masking. Significance codes: ***p < 0.001, *p < 0.05.

In quiet, all listeners scored at or near ceiling in the identification task (Figure 4A), consistent with previous results that nine-band speech stimuli remain highly intelligible despite vocoding (Shannon et al., 1995). Speech identification thresholds were much worse in SPEECH than NOISE thresholds (Figure 4B), confirming that the current stimulus processing produces IM (Arbogast et al., 2002). Using Bayesian inference, each listener's SPEECH and NOISE percent correct speech identification curves were fitted with sigmoidally shaped psychometric functions, as a function of TMR (Matlab toolbox: psignifit; Wichmann and Hill, 2001). Identification thresholds were defined as the TMR at 50% correct of these fitted functions. Paired *t*-tests comparing speech identification thresholds between SPEECH and NOISE found that performance was significantly worse in SPEECH [paired *t*-test, *t*_(10)_ = 25.4, *p* < 0.001]. The effect size, calculated as the Cohen's d ratio of the difference in SPEECH and NOISE thresholds divided by the pooled standard deviation across listeners, equaled 4.6. Similarly, speech keyword detectability was better in NOISE than SPEECH, by an average 0.4 d'-units [Figure 4C; paired *t*-test, *t*_(10)_ = –2.6, *p* = 0.027]. Cohen's d equaled 1.0.

We wished to eliminate the possibility of artifacts from eye movements and visual attention in our hemodynamic traces. Moreover, we wished to have comparable task difficulty across the tested conditions with fNIRS. Therefore, we next selected the keyword detection task for neuroimaging, because listeners could perform it with minimal body movement and closed eyes. Moreover, task performance was more comparable across maskers for speech detection vs. the identification task.

### 4.8. Neuroimaging Procedure

For both experiments, each listener completed one session of behavioral testing while we simultaneously recorded bilateral hemodynamic traces in the vicinity of STG and cIFS, using fNIRS. Throughout testing listeners held their eyes closed. Traces were acquired in 23-min sessions, consisting of 11 blocks of a controlled breathing task (9 min), followed by a brief break (ca. 2 min) and twelve blocks of auditory assessment (12 min). The controlled breathing task was identical to our prior methods [see details in Zhang et al. (2018)]. Briefly, the task consisted of 11 45-s-long blocks. In each block, listeners were instructed to breathe in for 5 se breathe out again for 5 s. This breathe-in-breathe-out pattern repeated for 6 times (30 s in total) before the listeners were instructed to hold breath for 15 s. The hemodynamic traces collected during this task establish a baseline dynamic range, from baseline to saturation, over which the optical recordings could vary for each particular listener, recording day and ROI. The auditory assessment was the behavioral detection task described above (see Behavioral Pilot Control).

### 4.9. Recording Setup for fNIRS

The listener wore insert earphones (Etymotic Research ER-2) and a custom-made fNIRS head-cap and held a wireless response interface in the lap (Microsoft Xbox 360 Wireless Controller; Figure 1A). Acoustic stimuli were generated on a laptop (Lenovo ThinkPad T440P) with Matlab (Release R2016a, The Mathworks, Inc., Natick, MA, USA), D/A converted with a sound card (Emotiva Stealth DC-1; 16 bit resolution, 44.1 kHz sampling frequency) and presented over the insert earphones. This acoustic setup was calibrated with a 2-cc coupler, 1/2" pressure-field microphone and a sound level meter (Bruel&Kjaer 2250-G4). The testing suite had intermittent background sound level with peak levels of 44 dBA (moderately quiet university hallway with noise from staff walking by). Together with the ER-2 insert earphones, which provide approximately 30 dB attenuation, the effective background noise level reaching the listener's tympanic membrane was 14 dB A, i.e., moderately quiet.

A camera-based 3D-location tracking and pointer tool system (Brainsight 2.0 software and hardware by Rogue Research Inc., Canada) was used to place the optodes above the left and right cIFS and STG, referenced to standardized brain coordinates (Talairach Atlas; Lancaster et al., 2000). A custom-built head cap, fitted to the listener's head via adjustable straps, embedded the optodes and held them in place.

Hemodynamic traces were recorded with a 4-source and 16-detector continuous-wave fNIRS system (690 and 830 nm optical wavelengths, 50 Hz sampling frequency; CW6, TechEn Inc.). The system therefore limited us to 2 sources and 8 detectors on each side of the head. The spatial layout of the optical source-detector pairs was custom-designed to cover each of the four ROIs cross-wise using deep channels with source-detector distances of 3 cm (solid lines in the bottom insert in Figure 1A) and one short separation channel with a source-detector distance of 1.5 cm (dashed lines in bottom insert of Figure 1A). Specifically, on each side of the head, leveraging time-multiplexing, two of the detectors were used for both sources—alternating between serving as a short vs. a deep channel (denoted by the blue dots near the center of the bottom of Figure 1A). For each of the resulting 16 deep and 4 shallow source-detector pairs, we then used simulated photon paths to estimate a sensitivity map across the surface of brain by mapping the light paths through a standardized head (Figure 1C, AtlasViewer; Aasted et al., 2015).

### 4.10. Signal Processing of the fNIRS Traces

Raw fNIRS traces were processed to estimate hemodynamic activation strength (Figure 1A and Supplemental Information 2). We first used HOMER2 to process the raw recordings during both the breath holding and auditory tasks, at each of the 16 deep and four shallow source-detector channels (Huppert et al., 2009). Specifically, the raw recordings were band-pass filtered between 0.01 and 0.1 Hz, using time-reversal filtering with a fifth order zero-phase Butterworth filter for high pass filtering and time-reversal filtering with a third order zero-phase Butterworth filter for low pass filtering (commands *filtfilt* and *butter* in Matlab 2016). Next, we removed slow temporal drifts in the band-pass filtered traces by de-trending each trace with a 20th-degree polynomial (Pei et al., 2007). To suppress artifacts due to sudden head movement, these de-trended traces were then transformed with Daubechies-2 base wavelet functions. Wavelet coefficients outside the one interquartile range were removed, before the remaining coefficients were inversely transformed (Molavi and Dumont, 2012). We then applied a modified Beer-Lambert law to these processed traces, resulting in the estimated oxygenated hemoglobin (HbO) and deoxygenated hemoglobin (HbR) concentrations for each channel (Cope and Delpy, 1988; Kocsis et al., 2006). To obtain hemoglobin changes relative to the maximum dynamic recording range for each individual listener and recording site, we then applied a normalization step. Specifically, for each listener and each of the 20 source-detector channels, we divided the HbO and HbR concentration from the task conditions by the peak of the HbO concentration change during the controlled breathing task, resulting in normalized HbO and HbR traces for each channel. Finally, we averaged the four deep channels at each ROI, resulting in a total of four task-evoked raw hemoglobin traces per ROI and listener (deep and shallow, HbO and HbR). We previously found that this dynamic range normalization step helps reduce across-listener variability in our listener population with a diverse range of skin pigmentations, hair consistencies and skull thicknesses (Zhang et al., 2018).

### 4.11. Hemodynamic Activation

To estimate auditory-task-evoked neural activity predicted by fixed effects of high- vs. low-IM, for each of the two experiments, we next fitted a linear mixed effect model (LMEM) to the pre-processed deep HbO and HbR traces (see Supplemental Information 2 for details on the equations). The LMEM model assumes that three main sources of variance shape the HbO and HbR traces: (1) a task-evoked response with IM independence (significant task-evoked activation that does not covary with IM vulnerability), (2) a task-evoked response with IM dependence (significant task-evoked activation that covaries with IM vulnerability), and (3) nuisance signals, deemed to be unlikely of neural origin. In addition, the LMEM includes the following factors that are known to drive neural response changes in STG and cIFS: audibility as modeled through left and right across-frequency average PTAs, and plasticity as modeled through change in output attributed to block number. To allow direct comparison of the masker evoked responses across different ROIs, all β_*i*_ were referenced relative to the SPEECH recordings in left cIFS.

To estimate whether a neural response captures behavioral phenotypes for vulnerability to IM, for each listener, masker configuration and ROI, we calculated the predicted total HbO and HbR responses from the LMEM weights, ignoring nuisance signals, PTA and plasticity. We next identified when the reconstructed HbO or HbR traces reached their maxima during the task interval, and measured the amplitudes at those single time points. Using these peak height of the reconstructed HbO or HbR traces as a measure of that ROI's neural recruitment for that masker, we then evaluated whether that ROI's hemodynamic recruitment correlated with the listener's behavioral d' sensitivity to IM.

## Data Availability Statement

The raw data supporting the conclusions of this article will be made available by the authors, without undue reservation.

## Ethics Statement

The studies involving human participants were reviewed and approved by Institutional Review Board of the New Jersey Institute of Technology. The patients/participants provided their written informed consent to participate in this study.

## Author Contributions

AI and MZ designed the experiments. MZ, AI, and NA implemented the experiment. MZ collected the data. MZ, AI, and NA analyzed the data. AI wrote the manuscript. All authors contributed to the article and approved the submitted version.

## Conflict of Interest

The authors declare that the research was conducted in the absence of any commercial or financial relationships that could be construed as a potential conflict of interest.

## References

[B1] Aasted C. M. Yücel M. A. Cooper R. J. Dubb J. Tsuzuki D. Becerra L.. (2015). Anatomical guidance for functional near-infrared spectroscopy: Atlasviewer tutorial. Neurophotonics 2:020801. 10.1117/1.NPh.2.2.020801 26157991 PMC4478785

[B2] Anderson C. A. Wiggins I. M. Kitterick P. T. Hartley D. E. (2017). Adaptive benefit of cross-modal plasticity following cochlear implantation in deaf adults. Proc. Natl. Acad. Sci. U.S.A. 114, 10256–10261. 10.1073/pnas.1704785114 28808014 PMC5617272

[B3] Arbogast T. L. Mason C. R. Kidd G. Jr (2002). The effect of spatial separation on informational and energetic masking of speech. J. Acoust. Soc. Am. 112, 2086–2098. 10.1121/1.1510141 12430820

[B4] Bharadwaj H. M. Khan S. Hämäläinen M. Kenet T. (2016). Electrophysiological correlates of auditory object binding with application to autism spectrum disorders. J. Acoust. Soc. Am. 140, 3045–3045. 10.1121/1.4969457

[B5] Bharadwaj H. M. Mai A. R. Simpson J. M. Choi I. Heinz M. G. Shinn-Cunningham B. G. (2019). Non-invasive assays of cochlear synaptopathy–candidates and considerations. Neuroscience 407, 53–66. 10.1016/j.neuroscience.2019.02.031 30853540 PMC6513698

[B6] Bouma H. (1970). Interaction effects in parafoveal letter recognition. Nature 226, 177–178. 10.1038/226177a0 5437004

[B7] Brungart D. S. (2001a). Evaluation of speech intelligibility with the coordinate response measure. J. Acoust. Soc. Am. 109, 2276–2279. 10.1121/1.1357812 11386582

[B8] Brungart D. S. (2001b). Informational and energetic masking effects in the perception of two simultaneous talkers. J. Acoust. Soc. Am. 109, 1101–1109. 10.1121/1.1345696 11303924

[B9] Brungart D. S. Chang P. S. Simpson B. D. Wang D. (2006). Isolating the energetic component of speech-on-speech masking with ideal time-frequency segregation. J. Acoust. Soc. Am. 120, 4007–4018. 10.1121/1.2363929 17225427

[B10] Brungart D. S. Simpson B. D. (2002). The effects of spatial separation in distance on the informational and energetic masking of a nearby speech signal. J. Acoust. Soc. Am. 112, 664–676. 10.1121/1.1490592 12186046

[B11] Brungart D. S. Simpson B. D. (2007). Effect of target-masker similarity on across-ear interference in a dichotic cocktail-party listening task. J. Acoust. Soc. Am. 122, 1724–1734. 10.1121/1.2756797 17927432

[B12] Chen Z. Hu G. Glasberg B. R. Moore B. C. (2011). A new method of calculating auditory excitation patterns and loudness for steady sounds. Hear. Res. 282, 204–215. 10.1016/j.heares.2011.08.001 21851853

[B13] Choi I. Wang L. Bharadwaj H. Shinn-Cunningham B. (2014). Individual differences in attentional modulation of cortical responses correlate with selective attention performance. Hear. Res. 314, 10–19. 10.1016/j.heares.2014.04.008 24821552 PMC4096237

[B14] Cope M. Delpy D. T. (1988). System for long-term measurement of cerebral blood and tissue oxygenation on newborn infants by near infra-red transillumination. Med. Biol. Eng. Comput. 26, 289–294. 10.1007/BF02447083 2855531

[B15] Davis M. H. Johnsrude I. S. (2003). Hierarchical processing in spoken language comprehension. J. Neurosci. 23, 3423–3431. 10.1523/JNEUROSCI.23-08-03423.2003 12716950 PMC6742313

[B16] Davis M. H. Johnsrude I. S. (2007). Hearing speech sounds: top-down influences on the interface between audition and speech perception. Hear. Res. 229, 132–147. 10.1016/j.heares.2007.01.014 17317056

[B17] Delgutte B. (1990). Physiological mechanisms of psychophysical masking: observations from auditory-nerve fibers. J. Acoust. Soc. Am. 87, 791–809. 10.1121/1.398891 2307776

[B18] Ding N. Simon J. Z. (2012). Emergence of neural encoding of auditory objects while listening to competing speakers. Proc. Natl. Acad. Sci. U.S.A. 109, 11854–11859. 10.1073/pnas.1205381109 22753470 PMC3406818

[B19] Durlach N. I. Mason C. R. Kidd G. Jr Arbogast T. L. Colburn H. S. Shinn-Cunningham B. G. (2003). Note on informational masking (l). J. Acoust. Soc. Am. 113, 2984–2987. 10.1121/1.1570435 12822768

[B20] Flom M. C. Heath G. G. Takahashi E. (1963). Contour interaction and visual resolution: contralateral effects. Science 142, 979–980. 10.1126/science.142.3594.979 14069233

[B21] Freyman R. L. Helfer K. S. McCall D. D. Clifton R. K. (1999). The role of perceived spatial separation in the unmasking of speech. J. Acoust. Soc. Am. 106, 3578–3588. 10.1121/1.428211 10615698

[B22] Füllgrabe C. Moore B. C. Stone M. A. (2015). Age-group differences in speech identification despite matched audiometrically normal hearing: contributions from auditory temporal processing and cognition. Front. Aging Neurosci. 6:347. 10.3389/fnagi.2014.00347 25628563 PMC4292733

[B23] Gallun F. J. Mason C. R. Kidd G. Jr (2005). Binaural release from informational masking in a speech identification task. J. Acoust. Soc. Am. 118, 1614–1625. 10.1121/1.1984876 16240821

[B24] Gallun F. J. Mason C. R. Kidd G. Jr (2007). The ability to listen with independent ears. J. Acoust. Soc. Am. 122, 2814–2825. 10.1121/1.2780143 18189571

[B25] Goupell M. J. Kan A. Litovsky R. Y. (2016). Spatial attention in bilateral cochlear-implant users. J. Acoust. Soc. Am. 140, 1652–1662. 10.1121/1.4962378 27914414 PMC5848865

[B26] Greenwood D. D. (1990). A cochlear frequency-position function for several species—29 years later. J. Acoust. Soc. Am. 87, 2592–2605. 10.1121/1.399052 2373794

[B27] Gutschalk A. Micheyl C. Oxenham A. J. (2008). Neural correlates of auditory perceptual awareness under informational masking. PLoS Biol. 6:e138. 10.1371/journal.pbio.0060138 18547141 PMC2422852

[B28] Hejna D. Musicus B. R. (1991). The solafs time-scale modification algorithm, in Bolt, Beranek and Newman (BBN) Technical Report.

[B29] Hofmann M. Wouters J. (2010). Electrically evoked auditory steady state responses in cochlear implant users. J. Assoc. Res. Otolaryngol. 11, 267–282. 10.1007/s10162-009-0201-z 20033246 PMC2862921

[B30] Huppert T. J. Diamond S. G. Franceschini M. A. Boas D. A. (2009). Homer: a review of time-series analysis methods for near-infrared spectroscopy of the brain. Appl. Optics 48, D280–D298. 10.1364/AO.48.00D280 19340120 PMC2761652

[B31] Ihlefeld A. Shinn-Cunningham B. (2008). Spatial release from energetic and informational masking in a selective speech identification task. J. Acoust. Soc. Am. 123, 4369–4379. 10.1121/1.2904826 18537388 PMC9014252

[B32] Jamadar S. D. (2020). The crunch model does not account for load-dependent changes in visuospatial working memory in older adults. Neuropsychologia 142:107446. 10.1016/j.neuropsychologia.2020.107446 32234498

[B33] Kerlin J. R. Shahin A. J. Miller L. M. (2010). Attentional gain control of ongoing cortical speech representations in a “cocktail party”. J. Neurosci. 30, 620–628. 10.1523/JNEUROSCI.3631-09.2010 20071526 PMC2832933

[B34] Keshishian M. Akbari H. Khalighinejad B. Herrero J. L. Mehta A. D. Mesgarani N. (2020). Estimating and interpreting nonlinear receptive field of sensory neural responses with deep neural network models. Elife 9:e53445. 10.7554/eLife.53445 32589140 PMC7347387

[B35] Khalighinejad B. Herrero J. L. Mehta A. D. Mesgarani N. (2019). Adaptation of the human auditory cortex to changing background noise. Nat. Commun. 10, 1–11. 10.1038/s41467-019-10611-4 31175304 PMC6555798

[B36] Kidd G. Jr Best V. Mason C. R. (2008). Listening to every other word: examining the strength of linkage variables in forming streams of speech. J. Acoust. Soc. Am. 124, 3793–3802. 10.1121/1.2998980 19206805 PMC2676624

[B37] Kidd G. Jr Mason C. R. Arbogast T. L. Brungart D. S. Simpson B. D. (2003). Informational masking caused by contralateral stimulation. J. Acoust. Soc. Am. 113, 1594–1603. 10.1121/1.1547440 12656394

[B38] Klein S. A. (2001). Measuring, estimating, and understanding the psychometric function: a commentary. Percept. Psychophys. 63, 1421–1455. 10.3758/BF03194552 11800466

[B39] Kocsis L. Herman P. Eke A. (2006). The modified beer–lambert law revisited. Phys. Medi. Biol. 51:N91. 10.1088/0031-9155/51/5/N02 16481677

[B40] Lancaster J. L. Woldorff M. G. Parsons L. M. Liotti M. Freitas C. S. Rainey L.. (2000). Automated talairach atlas labels for functional brain mapping. Hum. Brain Mapp. 10, 120–131. 10.1002/1097-0193(200007)10:3<120::AID-HBM30>3.0.CO;2-8 10912591 PMC6871915

[B41] Lawrence R. J. Wiggins I. M. Anderson C. A. Davies-Thompson J. Hartley D. E. (2018). Cortical correlates of speech intelligibility measured using functional near-infrared spectroscopy (fnirs). Hear. Res. 370, 53–64. 10.1016/j.heares.2018.09.005 30292959

[B42] Lee A. K. Rajaram S. Xia J. Bharadwaj H. Larson E. Hämäläinen M.. (2013). Auditory selective attention reveals preparatory activity in different cortical regions for selection based on source location and source pitch. Front. Neurosci. 6:190. 10.3389/fnins.2012.00190 23335874 PMC3538445

[B43] Lorenzi C. Gilbert G. Carn H. Garnier S. Moore B. C. (2006). Speech perception problems of the hearing impaired reflect inability to use temporal fine structure. Proc. Natl. Acad. Sci. U.S.A. 103, 18866–18869. 10.1073/pnas.0607364103 17116863 PMC1693753

[B44] Lutfi R. A. Gilbertson L. Heo I. Chang A.-C. Stamas J. (2013). The information-divergence hypothesis of informational masking. J. Acoust. Soc. Am. 134, 2160–2170. 10.1121/1.4817875 23967946 PMC3765281

[B45] Mattys S. L. Davis M. H. Bradlow A. R. Scott S. K. (2012). Speech recognition in adverse conditions: a review. Lang. Cogn. Proc. 27, 953–978. 10.1080/01690965.2012.705006

[B46] Mesgarani N. Chang E. F. (2012). Selective cortical representation of attended speaker in multi-talker speech perception. Nature 485, 233–236. 10.1038/nature11020 22522927 PMC3870007

[B47] Michalka S. W. Kong L. Rosen M. L. Shinn-Cunningham B. G. Somers D. C. (2015). Short-term memory for space and time flexibly recruit complementary sensory-biased frontal lobe attention networks. Neuron 87, 882–892. 10.1016/j.neuron.2015.07.028 26291168 PMC4545499

[B48] Millin R. Arman A. C. Chung S. T. Tjan B. S. (2014). Visual crowding in v1. Cereb. Cortex 24, 3107–3115. 10.1093/cercor/bht159 23833128 PMC4224237

[B49] Molavi B. Dumont G. A. (2012). Wavelet-based motion artifact removal for functional near-infrared spectroscopy. Physiol. Measur. 33:259. 10.1088/0967-3334/33/2/259 22273765

[B50] Neff D. L. Dethlefs T. M. (1995). Individual differences in simultaneous masking with random-frequency, multicomponent maskers. J. Acoust. Soc. Am. 98, 125–134. 10.1121/1.413748 7608391

[B51] Neff D. L. Dethlefs T. M. Jesteadt W. (1993). Informational masking for multicomponent maskers with spectral gapsa. J. Acoust. Soc. Am. 94, 3112–3126. 10.1121/1.407217 8300950

[B52] Nourski K. V. Etler C. P. Brugge J. F. Oya H. Kawasaki H. Reale R. A.. (2013). Direct recordings from the auditory cortex in a cochlear implant user. J. Assoc. Res. Otolaryngol. 14, 435–450. 10.1007/s10162-013-0382-3 23519390 PMC3642273

[B53] Noyce A. L. Cestero N. Michalka S. W. Shinn-Cunningham B. G. Somers D. C. (2017). Sensory-biased and multiple-demand processing in human lateral frontal cortex. J. Neurosci. 37, 8755–8766. 10.1523/JNEUROSCI.0660-17.2017 28821668 PMC5588466

[B54] O'Sullivan J. A. Power A. J. Mesgarani N. Rajaram S. Foxe J. J. Shinn-Cunningham B. G.. (2015). Attentional selection in a cocktail party environment can be decoded from single-trial eeg. Cereb. Cortex 25, 1697–1706. 10.1093/cercor/bht355 24429136 PMC4481604

[B55] Oxenham A. J. Fligor B. J. Mason C. R. Kidd G. Jr (2003). Informational masking and musical training. J. Acoust. Soc. Am. 114, 1543–1549. 10.1121/1.1598197 14514207

[B56] Papesh M. A. Folmer R. L. Gallun F. J. (2017). Cortical measures of binaural processing predict spatial release from masking performance. Front. Hum. Neurosci. 11:124. 10.3389/fnhum.2017.00124 28377706 PMC5359282

[B57] Parthasarathy A. Hancock K. E. Bennett K. DeGruttola V. Polley D. B. (2020). Bottom-up and top-down neural signatures of disordered multi-talker speech perception in adults with normal hearing. Elife 9:e51419. 10.7554/eLife.51419 31961322 PMC6974362

[B58] Pei Y. Wang Z. Barbour R. L. (2007). NAVI-SciPort solution: a problem solving environment (PSE) for nirs data analysis, in Poster at Human Brain Mapping (Chicago, IL).

[B59] Pelli D. G. Palomares M. Majaj N. J. (2001). Crowding is unlike ordinary masking: distinguishing feature detection and integration. J. Vis. 4:12. 10.1167/4.12.12 15669917

[B60] Pichora-Fuller M. K. Kramer S. E. Eckert M. A. Edwards B. Hornsby B. W. Humes L. E.. (2016). Hearing impairment and cognitive energy: the framework for understanding effortful listening (fuel). Ear Hear. 37, 5S–27S. 10.1097/AUD.0000000000000312 27355771

[B61] Pollonini L. Olds C. Abaya H. Bortfeld H. Beauchamp M. S. Oghalai J. S. (2014). Auditory cortex activation to natural speech and simulated cochlear implant speech measured with functional near-infrared spectroscopy. Hear. Res. 309, 84–93. 10.1016/j.heares.2013.11.007 24342740 PMC3939048

[B62] Presacco A. Simon J. Z. Anderson S. (2016). Effect of informational content of noise on speech representation in the aging midbrain and cortex. J. Neurophysiol. 116, 2356–2367. 10.1152/jn.00373.2016 27605531 PMC5110638

[B63] Rönnberg J. Rudner M. Foo C. Lunner T. (2008). Cognition counts: a working memory system for ease of language understanding (elu). Int. J. Audiol. 47(Suppl. 2):S99–S105. 10.1080/14992020802301167 19012117

[B64] Rosen S. Chakravarthi R. Pelli D. G. (2014). The bouma law of crowding, revised: critical spacing is equal across parts, not objects. J. Vis. 14:10. 10.1167/14.6.10 25502230 PMC4527718

[B65] Rowland S. C. Hartley D. E. Wiggins I. M. (2018). Listening in naturalistic scenes: what can functional near-infrared spectroscopy and intersubject correlation analysis tell us about the underlying brain activity? Trends Hear. 22:2331216518804116. 10.1177/2331216518804116 30345888 PMC6198387

[B66] Ruggles D. Bharadwaj H. Shinn-Cunningham B. G. (2011). Normal hearing is not enough to guarantee robust encoding of suprathreshold features important in everyday communication. Proc. Natl. Acad. Sci. U.S.A. 108, 15516–15521. 10.1073/pnas.1108912108 21844339 PMC3174666

[B67] Scott S. K. Rosen S. Beaman C. P. Davis J. P. Wise R. J. (2009). The neural processing of masked speech: evidence for different mechanisms in the left and right temporal lobes. J. Acoust. Soc. Am. 125, 1737–1743. 10.1121/1.3050255 19275330

[B68] Scott S. K. Rosen S. Lang H. Wise R. J. (2006). Neural correlates of intelligibility in speech investigated with noise vocoded speech—positron emission tomography study. J. Acoust. Soc. Am. 120, 1075–1083. 10.1121/1.2216725 16938993

[B69] Scott S. K. Rosen S. Wickham L. Wise R. J. (2004). A positron emission tomography study of the neural basis of informational and energetic masking effects in speech perception. J. Acoust. Soc. Am. 115, 813–821. 10.1121/1.1639336 15000192

[B70] Shannon R. V. Zeng F.-G. Kamath V. Wygonski J. Ekelid M. (1995). Speech recognition with primarily temporal cues. Science 270, 303–304. 10.1126/science.270.5234.303 7569981

[B71] Stefanovic B. Warnking J. M. Pike G. B. (2004). Hemodynamic and metabolic responses to neuronal inhibition. Neuroimage 22, 771–778. 10.1016/j.neuroimage.2004.01.036 15193606

[B72] Taylor S. Brown D. (1972). Lateral visual masking: supraretinal effects when viewing linear arrays with unlimited viewing time. Percept. Psychophys. 12, 97–99. 10.3758/BF03212851

[B73] Tripathy S. P. Levi D. M. (1994). Long-range dichoptic interactions in the human visual cortex in the region corresponding to the blind spot. Vis. Res. 34, 1127–1138. 10.1016/0042-6989(94)90295-X 8184557

[B74] Vazquez A. L. Fukuda M. Kim S.-G. (2018). Inhibitory neuron activity contributions to hemodynamic responses and metabolic load examined using an inhibitory optogenetic mouse model. Cereb. Cortex 28, 4105–4119. 10.1093/cercor/bhy225 30215693 PMC6188559

[B75] Viswanathan V. Bharadwaj H. M. Shinn-Cunningham B. G. (2019). Electroencephalographic signatures of the neural representation of speech during selective attention. Eneuro 6:ENEURO.0057-19.2019. 10.1523/ENEURO.0057-19.2019 31585928 PMC6873161

[B76] Wichmann F. A. Hill N. J. (2001). The psychometric function: I. fitting, sampling, and goodness of fit. Percept. Psychophys. 63, 1293–1313. 10.3758/BF03194544 11800458

[B77] Wightman F. L. Kistler D. J. (2005). Informational masking of speech in children: Effects of ipsilateral and contralateral distracters. J. Acoust. Soc. Am. 118, 3164–3176. 10.1121/1.2082567 16334898 PMC2819474

[B78] Wightman F. L. Kistler D. J. O'Bryan A. (2010). Individual differences and age effects in a dichotic informational masking paradigm. J. Acoust. Soc. Am. 128, 270–279. 10.1121/1.3436536 20649222 PMC2921429

[B79] Wijayasiri P. Hartley D. E. Wiggins I. M. (2017). Brain activity underlying the recovery of meaning from degraded speech: a functional near-infrared spectroscopy (fnirs) study. Hear. Res. 351, 55–67. 10.1016/j.heares.2017.05.010 28571617

[B80] Wild C. J. Davis M. H. Johnsrude I. S. (2012). Human auditory cortex is sensitive to the perceived clarity of speech. Neuroimage 60, 1490–1502. 10.1016/j.neuroimage.2012.01.035 22248574

[B81] Young E. D. Barta P. E. (1986). Rate responses of auditory nerve fibers to tones in noise near masked threshold. J. Acoust. Soc. Am. 79, 426–442. 10.1121/1.393530 3950195

[B82] Zekveld A. A. Kramer S. E. (2014). Cognitive processing load across a wide range of listening conditions: Insights from pupillometry. Psychophysiology 51, 277–284. 10.1111/psyp.12151 24506437

[B83] Zhang M. Alamatsaz N. Ihlefeld A. (2021). Hemodynamic responses link individual differences in informational masking to the vicinity of superior temporal gyrus. Dryad [Dataset]. 10.5061/dryad.gxd2547m6 PMC8339305 34366772

[B84] Zhang M. Mary Ying Y.-L. Ihlefeld A. (2018). Spatial release from informational masking: evidence from functional near infrared spectroscopy. Trends Hear. 22:2331216518817464. 10.1177/2331216518817464 30558491 PMC6299332

[B85] Zhou J. Benson N. Winawer J. Pelli D. (2018a). Conservation of crowding distance in human v4. J. Vis. 18, 856–856. 10.1167/18.10.856

[B86] Zhou X. Seghouane A.-K. Shah A. Innes-Brown H. Cross W. Litovsky R.. (2018b). Cortical speech processing in postlingually deaf adult cochlear implant users, as revealed by functional near-infrared spectroscopy. Trends Hear. 22:2331216518786850. 10.1177/2331216518786850 30022732 PMC6053859

