# Supplementary material for: Hemodynamic Responses Link Individual Differences in Informational Masking to the Vicinity of Superior Temporal Gyrus
Source: Front Neurosci. 2021 Jul 22;15:675326. doi: 10.3389/fnins.2021.675326 (PMC8339305; doi:10.3389/fnins.2021.675326)
Supplement: Supplementary file 1 [file Presentation_1.pdf]

Supplemental Information 1

Differences between behavioral pilot vs. fNIRS testing

During behavioral pilot testing, a significant but small effect of masker emerged in the speech detection task. However, during fNIRS testing, any differences between SPEECH vs. NOISE in the same behavioral task were too small to reach statistical significance. Specifically, averaged across listeners, speech detection performance in SPEECH equaled 1.97 (S.E. 0.11) during pilot testing as compared to 1.26 (S.E. 0.22) in experiment 1 and 1.71 (S.E. 0.16) in experiment 2. Across-listener average speech detection performance in NOISE equaled 2.41 (S.E. 0.13) during pilot testing vs. 1.56 (S.E. 0.21) in experiment 1.

The acoustic delivery of stimuli was identical for fNIRS testing and the behavioral pilot, except that testing happened in different rooms. The fNIRS testing suite had environmental background sound, but it was modest. Indeed, the energy reaching the ears from environmental sound in the fNIRS suite was 50 dB softer than either the masker or target source, as calibrated with a KEMAR acoustic manikin, presumably only subtly worsening EM or not at all, as compared to the behavioral pilot suite. This hints that the overall reduced performance during fNIRS testing is due to listeners being either more distracted and/or having to put more effort into performing the behavioral task when wearing fNIRS head caps.

Supplemental Information 2

LMEM

For each experiment, listener and source-detector pair, full hemodynamic traces were pre-processed (Supplemental Information 2 Figure 1A) before task-evoked responses were estimated *via* LMEM (Supplemental Information 2 Figure 1B).

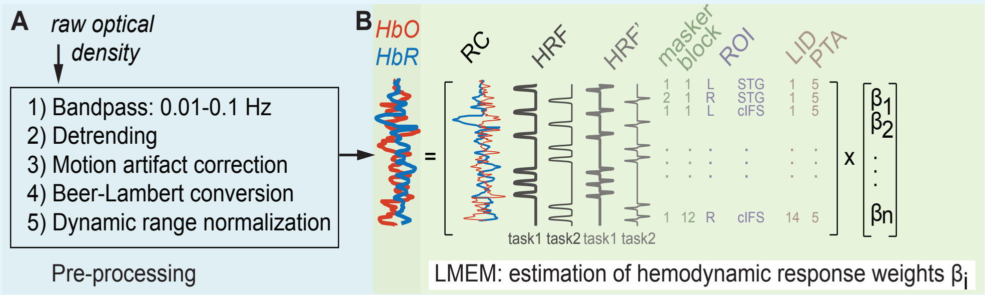

Supplemental Information 2 Figure 1. For each experiment, all recorded traces were fit with one LMEM. **(A)** Signal pre-processing steps. **(B)** Illustration of default effects in the LMEM.

The hemodynamic response function (HRF, ***Lindquist et al.*** (***2009***) is described by:

$$\text{HRF(t)} = \frac{1}{\Gamma(6)} t^5 e^{-t} - \frac{1}{6\Gamma(16)} t^1 5 e^{-t}$$

A single LMEM per experiment then fitted these normalized full HbO and HbR traces as follows:

HG_d = (Intercept $\cdot\beta_0$+ HRF$_{HbO}\cdot\beta_1$+ HRF'$_{HbO}\cdot\beta_2$+ #default effects
HRF$_{HbR}$ $\cdot\beta_3$+ HRF'$_{HbR}\cdot\beta_4$+ Block number $\cdot\beta_5$+
Reference channel$_{HbO}\cdot\beta_6$+ Reference channel$_{HbR}$ $\cdot\beta_7$+
Hemisphere $\cdot\beta_8$+ Cortical structure$\cdot\beta_9$+ Masker configuration $\cdot\beta_{10}$+
R Audio threshold $\cdot\beta_{11}$+ L Audio threshold $\cdot\beta_{12}$)+

(Cortical structure : Masker configuration $\cdot\beta_{13}$+ # interactions
Hemisphere : Masker configuration $\cdot\beta_{14}$+
Hemisphere : Cortical structure $\cdot\beta_{15}$+
HbO HRF : Masker configuration $\cdot\beta_{16}$+
HbO HRF : Cortical structure $\cdot\beta_{17}$+
HbO HRF : Hemisphere $\cdot\beta_{18}$+
HbO HRF' : Masker configuration $\cdot\beta_{19}$+
HbO HRF' : Cortical structure $\cdot\beta_{20}$+
HbO HRF': Hemisphere $\cdot\beta_{21}$+
HbR HRF : Masker configuration $\cdot\beta_{22}$+
HbR HRF : Cortical structure $\cdot\beta_{23}$+
HbR HRF : Hemisphere $\cdot\beta_{24}$+
HbR HRF' : Masker configuration $\cdot\beta_{25}$+
HbR HRF' : Cortical structure $\cdot\beta_{26}$+
HbR HRF' : Hemisphere $\cdot\beta_{27}$+
listener-dependent (Task condition + Cortical Structure + Hemisphere) # random effects

where HG_d is a two-dimensional vector of the normalized full hemoglobin concentrations, HbO and HbR, recorded from the deep source-detector channels. The β_i weights as-

sociated with each term are a linear measure of how much the term affected the recorded hemoglobin concentration change, relative to the reference condition of SPEECH in left cIFS.

To adjust the onset of the fitted functions to each individual, the LMEM included HRF', the first derivative of HRF (***Uga et al., 2014***).

Moreover, the LMEM default effects Hemisphere, Cortical structure, and Masker configuration each were two-level categorical variables representing two hemispheres (left vs. right, β_8), two cortical structures (cIFS vs. STG, β_9) and two task conditions per experiment (SPEECH vs. NOISE in experiment 1; SPEECH vs. SPEECH-OPPO in experiment 2, β_{10}). Together, these default effects estimated task-evoked responses in the HbO and HbR traces. In addition, the LMEM included factors that are known to drive neural response changes in STG and cIFS: plasticity, modelled through block number (β_5), as well as peripheral hearing, modelled through each individual listener's across-frequency average left and right PTA (β_{12} and β_{13}). Finally, cardiovascular nuisance signals unlikely to be of neural origin were regressed out via the shallow source-detector Reference channels (RC; β_{6-7}) in the default model.

As a result, this LMEM implicitly considered that HbR hemodynamic responses are generally much smaller in amplitude and build up more slowly, as compared to HbO (***Watanabe et al., 1996***; ***Sato et al., 2004***). Specifically, HRFs for HbR and HbO were of the same overall canonical functional form. However, to capture potentially different amplitudes and temporal onsets of HbO and HbR, the LMEM fitted HRF and HRF' amplitudes and their interactions with Masker configuration, Hemisphere and Cortical structure separately for HbO vs. HbR (β_{1-4};(***Niioka et al., 2018***)).

Finally, to regress out idiosynchratic listener-dependent effects on HbO and HbR traces (***Sato et al., 2005***; ***Minati et al., 2011***), the LMEM included random effects for each listener of Masker configuration, Cortical structure and Hemisphere. Note that we initially explored a range of statistical models. We deemed this LMEM model best in terms of explanatory power and parsimony, because it yielded low overall Akaike's Information Criterion and Bayesian Information Criterion scores (***Anderson and Burnham, 2002***).

Supplemental Information 3

Temporal Buildup

Using PET, prior work discovered stronger bilateral STG activation for speech masked by speech relatively to a speech masked by speech baseline (**?**), a finding confirmed by the current results via fNIRS. That prior work assessed speech identification while participants listened passively (**?**). In contrast, here, hemodynamic responses were recorded while listeners were actively engaged in a speech detection task. Of note, the prior study also showed that the left STG was more strongly recruited than right STG under IM (**?**). To examine hemispheric differences, we compared the LMEM predicted hemodynamic response across left and right hemisphere for STG, and, separately for STG. However, for the stimuli tested here during active listening, no robust hemispheric differences in STG activation were revealed.

Furthermore, frontal cortex peak responses during an EM task were found to lag behind STG responses, by approximately 1.5 seconds, when normally-hearing listeners were assessed with fNIRS while listening to vocoded speech in noise (**?**). To analyze the temporal buildup of the task-evoked responses, we subtracted the responses attributed by the LMEM to the STG from those attributed to the cIFS.

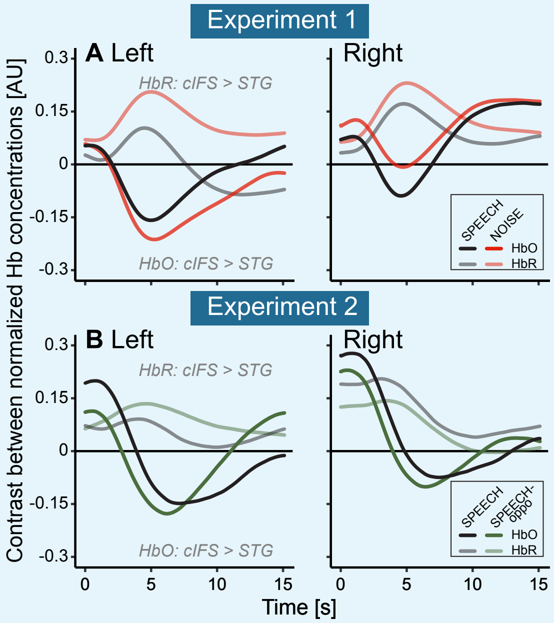

Supplemental Information 3 Figure 1. For the first 2-5 seconds of the task, STG was more active than cIFS, whereas cIFS responded more strongly afterwards, with both regions being balanced in their relative activations near the end of the task interval (15 s). This temporal buildup was observed in both hemispheres, for all tested masker configurations, and for both HbO and HbR. Note that HbO (darker lines) and HbR (lighter lines) are anti-correlated, here, as expected. **(A)** Relative to each ROIs own peak activation levels, STG is slightly more strongly activated than cIFS, for both SPEECH (black) and NOISE in experiment 1(red), in both the Left and the Right hemisphere. **(B)** The temporal buildup of dominant STG vs. cIFS activity in experiment 2 are qualitatively comparable to the results in experiment 1 (compare black lines in top vs. bottom plots. Moreover, the pattern where early STG activity emerges prior to stronger cIFS recruitment also holds for SPEECH-oppo.

In both experiments, masker-evoked differences in overall recruitment of STG vs. cIFS varied over time. In experiment 1, STG was slightly more strongly recruited during the first 2 seconds of the task interval, before the recruitment between STG and cIFS became more balanced, in both the left and right hemispheres (Supplemental Information 3 Figure 1A). Similarly, in experiment 2, within each hemisphere, STG was relatively more engaged than

cIFS during the first 2-5 seconds of the task , followed by stronger HbO and HbR recruitment in the cIFS region (Supplemental Information 3 Figure 1B). Thus, the current temporal buildup results are consistent with prior findings that STG activates before frontal regions, for both EM and IM (**?**).

Supplemental Information 4

Breath Hold Normalization

The recorded concentration of HbO and HbR depends on how tightly optodes and skull are coupled. Here, optodes were mounted manually and adjusted via velcro-straps, raising the possibility that observed differences in overall observed activation strength across different ROIs could be contaminated by differences in optode-skull coupling. To control for this potential caveat, we previously developed a breath-holding normalization, illustrated in Figure 1. Specifically, assuming that cerebrovascular reactivity to hypercapnia during breath-holding is systemic and therefore comparable across the four ROIs, we normalize the HbO and HbR traces within each ROI by that ROI's HbO peak value during hypercapnia $|\hat{p}|$. The normalized responses factor out idiosyncratic differences across ROIs. Therefore, they can then be compared directly via LMEM. Pilot data (not shown) hint that this breath-holding normalization also reduces across-listener variability in the estimated task-evoked responses, at the population level. However, further testing is needed to confirm or reject this observation.

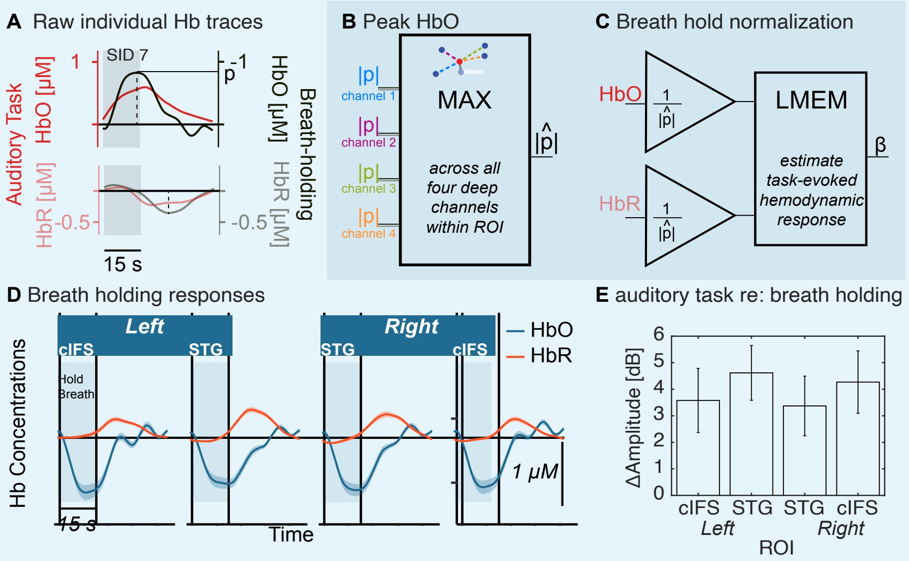

Supplemental Information 4 Figure 1. A Task-evoked and breath-holding-evoked hemodynamic responses for a representative listeners. **B** Within each of the four ROIs, peak HbO activation strength during breath holding is calculated for this ROI. **C** All HbO and HbR traces within each ROI are normalizaed by that ROI's overall HbO peak actication strength. **D** Raw across-listener average breath-holding HbO and HbR traces. Error ribbons show one standard error of the mean across listeners. **E** Across-listener average amplitude scaling factors. Task-evoked responses are approximately 4 dB smaller in amplitude as compared to breath-holding evoked responses, with idiosyncratic differences across ROIs. Error bars show one standard error of the mean across listeners.

Supplemental Information 5

Detailed Photon Path Simulations

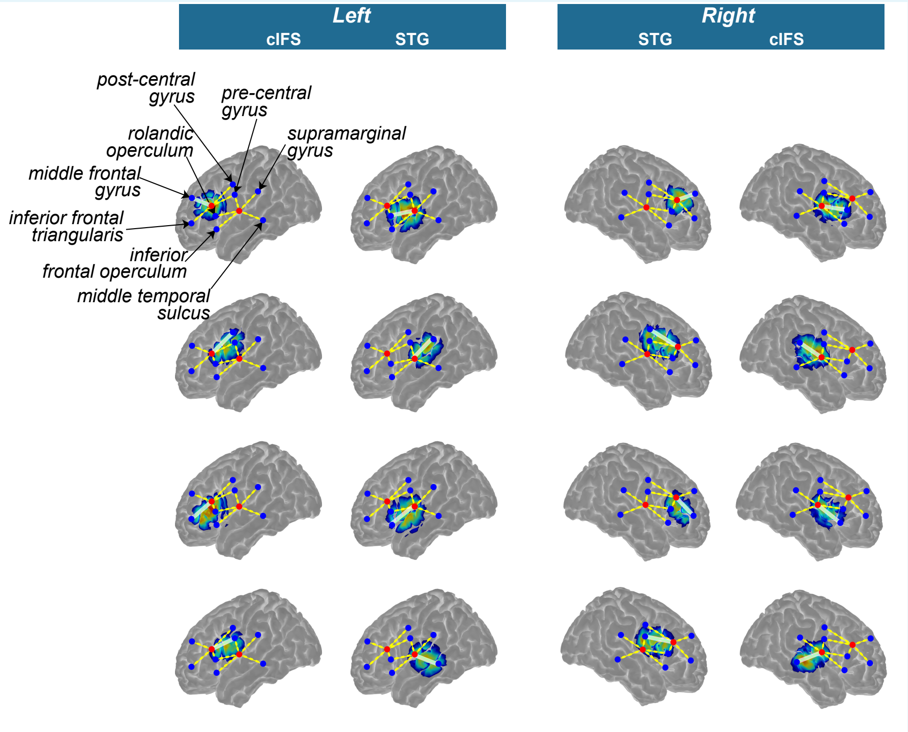

Supplemental Information 5 Figure 1. Simulated photon paths for each individual source-detector channel in each of the four ROIs confirm that the estimated recruited brain areas differ across channels and ROIs.

References

Anderson DR, Burnham KP. Avoiding pitfalls when using information-theoretic methods. The Journal of Wildlife Management. 2002; p. 912–918.

Lindquist MA, Loh JM, Atlas LY, Wager TD. Modeling the hemodynamic response function in fMRI: efficiency, bias and mis-modeling. Neuroimage. 2009; 45(1):S187–S198.

Minati L, Kress IU, Visani E, Medford N, Critchley HD. Intra-and extra-cranial effects of transient blood pressure changes on brain near-infrared spectroscopy (NIRS) measurements. Journal of neuroscience methods. 2011; 197(2):283–288.

Niioka K, Uga M, Nagata T, Tokuda T, Dan I, Ochi K. Cerebral hemodynamic response during concealment of information about a mock crime: Application of a general linear model with an adaptive hemodynamic response function. Japanese Psychological Research. 2018; 60(4):311–326.

Sato H, Fuchino Y, Kiguchi M, Katura T, Maki A, Yoro T, Koizumi H. Intersubject variability of near-infrared spectroscopy signals during sensorimotor cortex activation. Journal of biomedical optics. 2005; 10(4):044001.

Sato H, Kiguchi M, Kawaguchi F, Maki A. Practicality of wavelength selection to improve signal-to-noise ratio in near-infrared spectroscopy. Neuroimage. 2004; 21(4):1554–1562.

Uga M, Dan I, Sano T, Dan H, Watanabe E. Optimizing the general linear model for functional near-infrared spectroscopy: an adaptive hemodynamic response function approach. Neurophotonics. 2014; 1(1):015004.

Watanabe E, Yamashita Y, Maki A, Ito Y, Koizumi H. Non-invasive functional mapping with multi-channel near infra-red spectroscopic topography in humans. Neuroscience letters. 1996; 205(1):41–44.
